# The effects of graded levels of calorie restriction: IV. Non-linear change in behavioural phenotype of mice in response to short-term calorie restriction

**DOI:** 10.1038/srep13198

**Published:** 2015-08-25

**Authors:** David Lusseau, Sharon E. Mitchell, Ceres Barros, Davina Derous, Cara Green, Luonan Chen, Jing-Dong Jackie Han, Yingchun Wang, Daniel E. L. Promislow, Alex Douglas, John R. Speakman

**Affiliations:** 1 Institute of Biological and Environmental Sciences, University of Aberdeen, Aberdeen, Scotland, UK Ab24 2TZ; 2 Key laboratory of Systems Biology, Shanghai Institute of Biological Sciences, Chinese Academy of Sciences, 800 Cao Bao road, Shanghai, China; 3 Max Planck partner institute for computational biology, Shanghai; 4 State Key laboratory of Molecular Developmental Biology, Institute of Genetics and Developmental Biology, Chinese Academy of Sciences, 1 Beichen Xilu, Chaoyang, Beijing, China; 5 Department of Pathology, University of Washington at Seattle, Seattle, Washington, USA

## Abstract

Animals have to adjust their activities when faced with caloric restriction (CR) to deal with reduced energy intake. If CR is pronounced, allostasis can push individuals into alternate physiological states which can result in important health benefits across a wide range of taxa. Here we developed a new approach to determine the changes in behavioural phenotype associated with different levels of CR. We exposed C57BL/6 male mice to graded CR (from 0 to 40%) for three months and defined their behavioural phenotype using hidden Markov models of their movement and body temperature. All 40% CR mice exhibited a state-shift in behavioural phenotype and only some exposed to 30% CR did. We show for the first time that mice changed their activity characteristics rather than changed their activities. This new phenotyping approach provides an avenue to determine the mechanisms linking CR to healthspan.

Individuals need to maintain a certain activity budget to meet their physiological requirements – for example, to gather food or to find mates. There are a number of ways in which they can assort their activities during the day to meet this requirement, and therefore there are several potential assemblages of activities and behaviour that can be used to satisfy physiological needs^1^. Some constraints exist that will limit these behavioural dynamics. For example, digestive restrictions like stomach capacity, may limit feeding bout duration and predation risk may make foraging at given times unfeasible. In a natural environment, when and where opportunities and risks arise will constrain whether an individual can carry out some activities and for how long. State-dependent behavioural phenotypes will therefore emerge from selection on the complex interactions of intrinsic needs and environmental pressures^2^,^3^,^4^,^5^. We can therefore think of behavioural phenotypes as a way to describe the state-dependent way by which individuals organise their daily lives^6^. It is particularly important to recognize that the temporal dynamics emerging from these complex interactions is itself a key characteristic of behavioural phenotypes, yet this aspect has received little attention to date^5^. It is a key characteristic because activity bout durations encapsulate the constraints individuals face to cease or instigate given activities. To date, behavioural phenotyping has primarily been used as a way to diagnose the display of certain features of the central nervous system^7^. We have primarily looked for the presence of specific behaviours^8^, reactions to specific stimuli^9^, or performance on standardised tests^8^. Here we introduce a more holistic approach to behavioural phenotyping as a way to capture the way individuals spend their day to meet their physiological requirements when faced by a change in environmental conditions. Finding a way to capture succinctly activity state characteristics as well as their temporal dynamics provides a way to describe the state of the complex system of interactions between internal needs and external opportunities^10^. Doing so provides a mean to assess when an individual – the complex system – may shift from one state to another, therefore identifying state-dependent phenotypic state shifts^4^,^6^.

Studies on a wide range of taxa show that reducing food intake in controlled environments increases lifespan by decreasing the onset of age-related diseases^11^,^12^,^13^,^14^,^15^. We do not know the mechanism generating these benefits. We do know that this caloric restriction (CR thereafter) presents an energetic allostatic challenge and individuals will alter their activities to regain homeostasis^16^,^17^,^18^. However, when CR is pronounced individuals may no longer be able to achieve energy balance within a particular behavioural phenotype. Therefore, they might shift to another phenotype allowing them to redress the balance between energetic expenditure and the restricted energy intake they can achieve under CR. Such changes have been partially observed before with, for example, the onset of lowered body temperature associated with lowered activity levels^19^,^20^ and its extreme form – torpor – in rodents^19^,^20^.

To date, we do not have a comprehensive understanding of the variability in behavioural phenotypes observed when individuals are facing CR which is needed to start to assess whether those state-shifts are related to health benefits. We therefore need to better understand how individuals change their behavioural phenotypes to meet the CR challenge. We expect that, since CR is an added constraint on activities that costs energy, as the level of CR increases individuals should shift their phenotypic state converging towards similar activity characteristics which allow them to stay alive at the reduced level of intake. Given our understanding of the effect of CR, we expect behavioural phenotypic changes in mice will include a change in the temporal dynamics of activities, e.g. a decrease in bout duration of an active state^21^. We also expect a change in the activity budget with the appearance of a torpor state for those mice exposed to higher levels of CR^20^. We aim to test these predictions using a unique experiment during which the behavioural phenotype of the mice could be continuously assayed using novel analytical approaches.

## Model Development

### Hidden Markov model of behavioural phenotype

We exposed mice (n = 7–9 per group) to five different levels of CR: 0, 10, 20, 30 and 40% lower calories than their own individual intakes measured for two weeks prior to introducing the restricted diets. Individuals facing no CR could have *ad-libitum* access to food for 12H (12AL) or 24H (24AL) (see methods for details). Mice were housed individually and placed on antenna pads which allowed continuous triangulation of the position of the mice using surgically implanted transmitters (VitalViewTM telemetry and data acquisition system using a ER-4000 receiving platform (MiniMitter, OR, USA) (see Gamo, Troup *et al.* 2013 for details). From these transmitters, we were able to observe the number of movements the mice made at given intervals (here every 15 minutes) as well as concurrently collect body temperature observations over the same intervals (see methods for details).

In a given physiological state, a mouse would partition its behaviour in a series of activities and assort the time it spends in those different activities in order to meet the need of that state, given the environmental constraints (here CR) it faces. This hidden process can be defined by describing activity states and the temporal dynamics of transition among those states (ultimately informing the activity budget and bout durations the mouse achieves). We develop a hidden Markov model (HMM) to model this hidden process from observations on the mice. We assumed that the movement count and body temperature data were observations emanating from a mouse hidden activity state and that both variables could be used to define those states. Such modelling assumptions have been successful in many studies to describe and predict animal behaviour dynamics^22^,^23^. In its simplest form, we expected the activity budget of mice to be composed of two states (active and inactive) and that a third state would be present under severe CR: torpor. We expected mice in an active state to move more and have a slightly higher body temperature since movement generates heat^24^, mice in an inactive state to move less and have a slightly lower body temperature and mice in a torpor state to be even less active and display a large drop in body temperature.

The observations informed a multivariate normal mixture model of *n* activity states. The multivariate normal distributions were composed of two normal distributions, describing the log of movement count + 1 (M_t_) and the body temperature (T_t_) at time *t*:


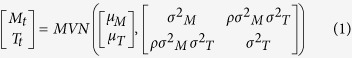


Here this distribution will depend on the activity state S_t_, where S_t_ = *i* and *i* ∈ [1, *n*], in which the individual finds itself. We are therefore fitting a mixture of multivariate normal distribution (MVN), each defined by a mean, μ_Mi_, and variance, σ^2^_Mi_, log movement count and a mean, μ_Ti_, and variance, σ^2^_Ti_, body temperature to describe the observed joint distribution of log movement count and body temperature. We assumed that M_t_ and T_t_ were conditionally independent given the states S_i,t_ so that we only estimated σ^2^_Mi_ and σ^2^_Ti_ (an assumption supported by post-hoc exploration for each state):


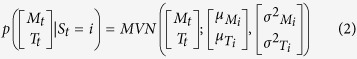


We assumed a Markovian process for the state transition for which we estimate the transition probability matrix (tpm) **P** defined as:


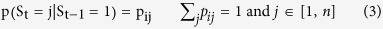


Therefore S_t_ depends not only on S_t-1_ but also M_t_ and T_t_ and so we estimated jointly the transition probability matrix between the n states and the mixture model parameters.

### Introducing the influence of covariates

This first model (HMM1) does not allow for any changes in the behavioural phenotype of a mouse throughout the experiment. We predicted that mice under greater CR would change their phenotypic state as they progress through the experiment. This could result in two temporal effects on HMM1: i) the characteristics of the multivariate normal distributions will change (Eq. 2), or ii) the dynamics of activity states will change; hence the transition probability matrix (Eq. 3) will change. We can foresee two factors that could cause such changes: the time elapsed since the CR exposure started (*t*) or the change in body weight (*BW*) caused by CR. *BW* and *t* are correlated^25^. To test for such effects we develop four alternative sets of models. The first set (HMM2) accounts for the effect of time elapsed, in 15-minute time steps, since the CR started on the MVN parameters:


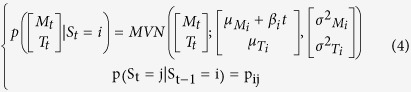



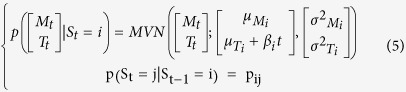



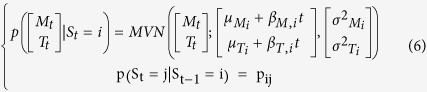


The second set of models (HMM3) accounts for the effect of BW, measured on each day *d*, on the MVN parameters:


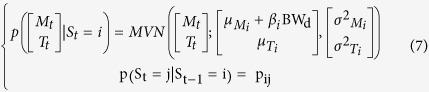



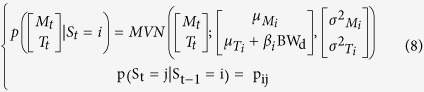



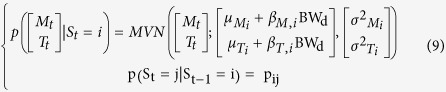


The third model (HMM4) accounts for the effect of time elapsed since the CR started on the state transition probability matrix:


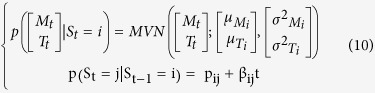


The last model (HMM5) accounts for the effect of daily BW on the transition probability matrix:


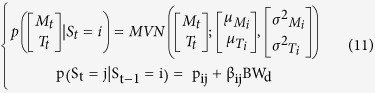


Finally, we also fitted a set of models that included effects on both the tpm and the MVN parameters (Table 1). All covariates were centred and standardised before being introduced in the models.

Ultimately we fitted 30 hidden Markov models to the observations of each mouse for both datasets: before CR treatment commenced (BL period) and under CR (Table 1). We decided to fit models to each mouse independently in order to determine whether mice from the same treatment levels behaved in a similar manner.

## Results

One individual (30CR) died of unknown cause during the experiment and was removed from these analyses.

### Model selection

The best models for all mice contained three activity states which could be characterised as relatively higher activity and higher body temperature (state 1), relatively lower activity (state 2) and relatively very low activity and lower body temperature (state 3) (Supplementary Table 2, Supplementary Figure 2). A three-state model also described best behaviour for the control period (Supplementary Table 1) as well as for the period during which mice were exposed to CR (Supplementary Table 2). Transition probabilities changed for many individuals throughout the two periods but not consistently with CR levels. However, the changes in characteristics of the activity states were consistent across all mice. While we fitted models independently to observations from each mouse, we found high level of consistency in the activity state characteristics within CR treatment levels. The proportion of individuals that changed their activity state characteristics as they changed BW (models from the HMM3 class, Table 1 and Supplementary Table 2) was larger for higher CR levels 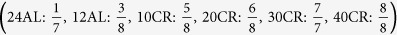.

### Activity state characteristics

**Figure 1:**
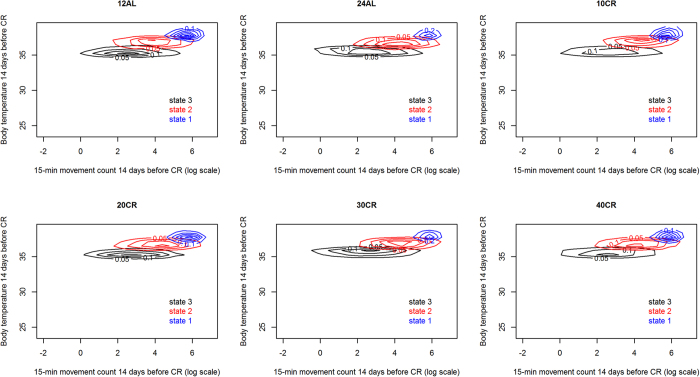
Fitted activity state characteristics for each treatment level at the start of the baseline period. The states are characterised by the log of the number of movements observed over a 15 minutes period and the median core body temperature of the mouse over those 15 minutes. Each colour represents a contour plot for a state (state 1 – blue, state 2 – red and state 3 – black) drawn from simulating 1000 observations for each mouse from its fitted HMM for each state. Simulated observations from all mice in a treatment level are cumulated in each contour plot. Note the high consistency within state between mice leading to well defined contours for each state.

Whilst the best fitting models included a change in activity state characteristics for the BL period, these changes were trivial (i.e. of little biological relevance as the effects are much smaller than the inherent variability in state characteristics) and did not differ between treatment levels (Fig. 1 and Supplementary Figure 1).

**Figure 2:**
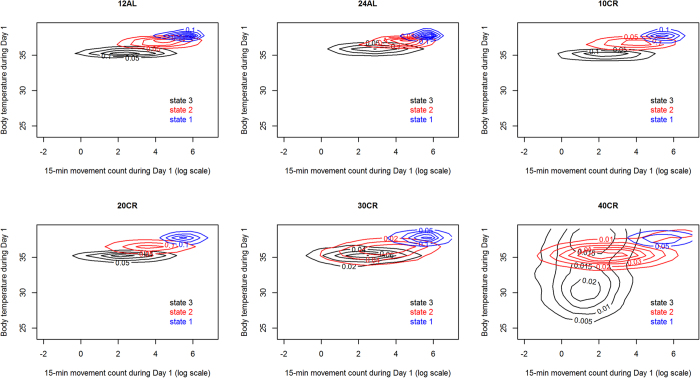
Fitted activity state characteristics for each treatment level at the start of the treatment period. The states are characterised by the log of the number of movements observed over a 15-minute period and the median core body temperature of the mouse over those 15 minutes. Each colour represents a contour plot for a state (state 1 – blue, state 2 – red and state 3 – black) drawn from simulating 1000 observations for each mouse from its fitted HMM for each state. Simulated observations from all mice in a treatment level are cumulated in each contour plot.

**Figure 3:**
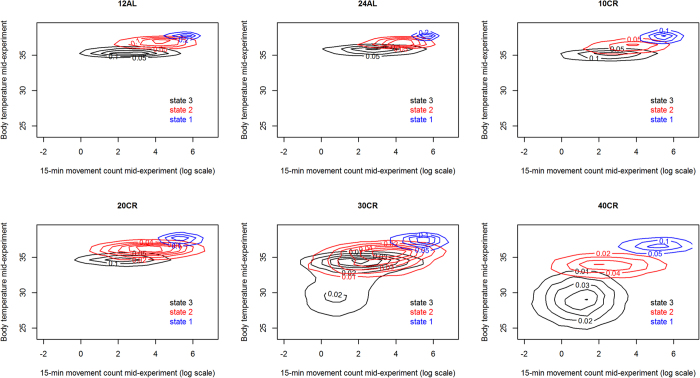
Fitted activity state characteristics for each treatment level halfway through the treatment period. The timing of this measure varied between individuals depending on the covariate in their fitted HMM (Supplementary Table 2). This means at day 40 for those individuals with a day covariate and halfway through body mass loss^25^ for those individuals with BW as a covariate. The states are characterised by the log of the number of movements observed over a 15-minute period and the median core body temperature of the mouse over those 15 minutes. Each colour represents a contour plot for a state (state 1 – blue, state 2 – red and state 3 – black) drawn from simulating 1000 observations for each mouse from its fitted HMM for each state. Simulated observations from all mice in a treatment level are cumulated in each contour plot.

**Figure 4:**
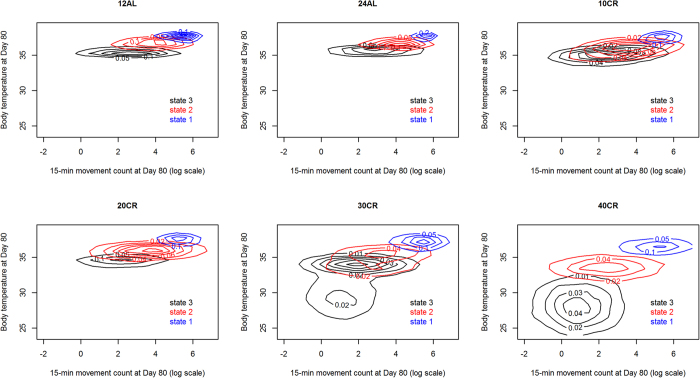
Fitted activity state characteristics for each treatment level at the end of the treatment period. The states are characterised by the log of the number of movements observed over a 15-minute period and the median core body temperature of the mouse over those 15 minutes. Each colour represents a contour plot for a state (state 1 – blue, state 2 – red and state 3 – black) drawn from simulating 1000 observations for each mouse from its fitted HMM for each state. Simulated observations from all mice in a treatment level are cumulated in each contour plot.

By contrast, we observed a non-linear change in behavioural phenotype as the CR treatment level increased (Figs 2, 3, 4). While the activity state characteristics for 10% and 20% CR remained essentially similar to the control levels (12AL and 24AL), those states progressively changed throughout the treatment period for 40% CR individuals and some 30% CR individuals (Fig. 4). We observed for both of those sets of individuals the emergence of torpor (Fig. 4, central tendency in body temperature dropping below 31 °C). However, and crucially, torpor here can be seen not as a new activity state emerging in those mice that require it, but as the culmination of a progressive change in the characteristics of state 3 (Figs 3 and 4).

**Figure 5:**
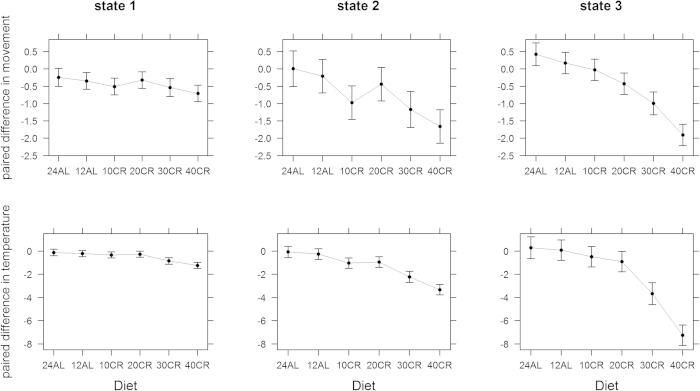
Fitted effect of diet on the paired difference in fitted characteristics of state 1, 2, and 3 activity states for individuals depending on their CR treatment level. Error bars are 95% confidence intervals determined from the three fitted multivariate linear models. A value of zero means that the characteristics did not change after CR treatment was introduced.

A paired comparison between the BL and the treatment periods shows that the changes we observed were not related to variability in behavioural phenotype among mice during BL. We fitted a multivariate linear model to the paired difference, by individuals, in state characteristics between the end of the CR treatment and the start of the BL period. CR affected the characteristics of state 1 (Pillai’s trace_5,40_ = 0.63, p = 0.0004, R^2^_adj_ = 0.52), state 2 (Pillai’s trace_5,40_ = 0.89, p < 0.0001, R^2^_adj_ = 0.76), and state 3 (Pillai’s trace_5,40_ = 1.06, p < 0.0001, R^2^_adj_ = 0.83). These effects are mostly driven by large changes for individuals exposed to 30% and 40% CR (Fig. 5). State 3 characteristics were significantly lowered for all levels of CR – albeit by small amounts for 10% and 20% CR (Fig. 5) which were not significantly different from the changes observed for the control group (12AL). Only 30% and 40% CR individuals had state characteristics that were significantly lower than 12AL for all states.

### Activity temporal dynamics

**Figure 6:**
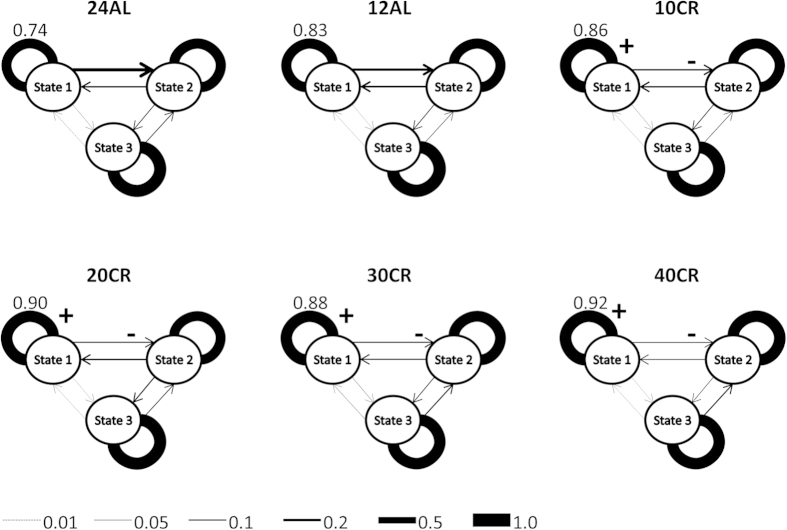
Diagram of the average transition probability matrices for each CR treatment level at the end of the CR treatment period (values are given for average p_11_). Transition probabilities that significantly decreased from end of the baseline period are marked with ‘−’ and those that significantly increased are marked with a ‘+’. Those represent a consistent tendency to change within a treatment level in contrast with BL conditions.

While the fitted models did not include changes in tpm for all individuals through the BL or the treatment periods (Supplementary Tables 1 & 2, Fig. 6), there were differences in the activity temporal dynamics linked with CR. The transitions from state 3 (Pillai’s trace_5,40_ = 0.43, p_adj_ = 0.22) and state 2 (Pillai’s trace_5,40_ = 0.28, p_adj_ > 0.5) did not change with CR, but transitions from state 1 (Pillai’s trace_5,40_ = 0.60, p_adj_ = 0.008) did. No significant changes were observed for the control groups (Fig. 6). All CR groups increased their probability to stay in a high activity state when they were in that state, an effect that was simply compensated by decreasing the probability to shift from state 1 to 2 (Fig. 6). The effect size increased with CR level leading to large changes in activity budget for this group (Supplementary Figure 3). It is important to note that, as expected, mice showed diurnal cycles in their activity (Supplementary Figure 3). This cyclicity, tuned by light/dark phases, was not incorporated in the model structure but rather emerged from the model fit. This provided another good measure of the goodness-of-fit of the models. The ‘day’ activity budget of mice was the most perturbed by 40% CR (Supplementary Figure 3).

## Discussion

We expected to see a change in the temporal dynamics of mouse activity as well as the emergence of a third activity state, torpor, in mice exposed to higher levels of CR. However, we observed that the transition probabilities among activity states did not always change throughout the CR treatment (Supplementary Table 2) and control mice were just as likely to change their transition probabilities as CR mice. Unexpectedly, we found that all mice had an activity budget composed of three states, even before CR treatment. We saw a consistent change in activity state characteristics that occurred as mice progressed through the treatment period. These changes were small for mice in both control levels and small CR levels (10% and 20%). Larger changes emerged in mice that had larger changes in their BW in response to CR and the pace of those changes was best explained by the time series of weight loss these mice incurred. As expected, the behavioural phenotype of mice became more constrained as their exposure to CR became more extreme: the same model explained behavioural phenotype in all 40% CR mice (Supplementary Table 2). The phenotypic changes we observed are consistent with previous studies: individuals exposed to CR respond by decreasing body temperature^20^. We show here for the first time though, that this change is not the same across the daily activities of mice. Some studies have observed mice to become more lethargic under CR^16^,^21^. What we show is that this lethargy might have been misinterpreted as a change in activity budget, when the mice are primarily changing their behavioural characteristics for each activity state. This is consistent with the observed decrease in energy spent on activity by other studies^18^. Mice will still display three activity levels but they will be performing less movement when CR changes their behavioural phenotype. The change in state characteristics was not consistent across all states. We see an order of magnitude of difference in the decrease in movement observed under severe CR if we compare state 1 to state 2. Previous work has also shown that torpor emerged to cope with CR^26^, but we show here that we can expect a consistent drop in body temperature throughout all activity states. Some evidence suggests that lowered body temperature may increase lifespan in mammals^27^. We can see then that under increasing CR, lowered body temperature throughout all activities could provide a mechanism for individuals to increase survival probability. This potential adaptive response would allow individuals to try and outlive CR condition and therefore attempt to maximise lifetime reproductive success.

Behavioural dynamics differed between mice from different CR treatment levels. As CR increased, mice had longer bouts of higher activity levels (increased p_11_). This change in activity transition probabilities occurred progressively (affected by BW or t, ESM1). This shift coincided with a progressive change in activity characteristics. These changes led to a change in activity budget in 40% CR mice. Other mice displayed the typical large shift in activity budget between light and dark phases, including food anticipatory activity before feeding^28^. By contrast, 40% CR mice spent more time in states 1 and 2 during the light phase. This is an important finding as it hints to the manner by which individuals change their behaviour to accommodate environmental variability. Even individuals exposed to extremely predictable environments, for several generations, can change not only the assortment of their activities, but also the way they carry out activities, to deal with environmental challenges. However, not all mice consistently changed their tpm throughout the treatment. This perhaps hints at activity state characteristics being the first mechanism individual can use to respond to environmental changes, rather than modifying their activity budget.

The differences we saw were not caused by pre-existing differences between mice but were the result of the CR treatment, as all mice had similar behavioural phenotypes before treatments. Beyond the study of the effect of CR on the body, this study poses interesting questions about the way individuals will shift their behaviour when faced with environmental challenges. We show that individuals can very quickly adapt the temporal dynamics of their activities to cope with such a challenge (Figs 1, 2, 3, 4). They do so here by increasing high activity bout duration probably to ensure the monopolisation of a scarce resource. However, this dynamic shift occurs in tandem with a more consistent change, across individuals, in the way they act to cope with an energetic challenge. This change in activity characteristics is likely to be driven by weight loss and is presumably aimed at conserving energy.

This study shows that there is a step change in the behavioural phenotype of mice at 30% CR, with all mice changing their phenotype at 40% CR and no mice changing it after 3 months at 20% CR. An exposure to 30% caloric restriction induced larger changes in the behavioural phenotype of some mice than in others. We now need to assess the differences between those 30% CR individuals at a molecular level to understand the mechanisms from which this variability emerges. We also now need to address whether the progressive change we observed in 30% and 40% CR might also emerge in lower levels of CR if exposure to CR is prolonged beyond three months. Indeed, 40% CR mice showed a rapid change in phenotype (Fig. 2). Progressive change in BW, and the organ-specific differential weight loss^25^ show that the duration of the CR challenge is going to be as much of an enactor of change as the CR level itself. What we can conclude is that after three months mice exposed to 40% CR will have changed their behavioural phenotype. Some mice exposed to 30% CR will do so also. This variability at 30% CR may just be the result of different pace at which individuals adapt to the energetic challenge. But it may also be a genuine threshold at which some individuals will see a radical change in body state, while others won’t. This may also dictate the pace at which the health benefits of CR may emerge or whether it will happen at all.

## Methods

### Overall design and rationale

All procedures were reviewed and approved by University of Aberdeen ethical approval committee and carried out under a Home Office issued license compliant with the Animals (Scientific Procedures) Act 1986. The methods were carried out in accordance with the approved guidelines.

C57BL/6 male mice were purchased from Charles River (Ormiston, UK). They were introduced to CR at 20 weeks of age, approximately equivalent to early human adulthood (see^25^ for further details). Previous studies suggested CR begun at 6 months was as effective at increasing lifespan as starting at 6 weeks^29^. We exposed mice (n = 7–9 per group) to five different levels of CR: 0, 10, 20, 30 and 40% lower calories than their own individual intakes measured for two weeks prior to introducing the restricted diets. Mice were randomly allocated into these six experimental groups and their body weight (BW) was recorded daily. Mice on restriction were individually housed and fed daily at lights out (1830 h). Because the mice adjusted their digestive efficiency, realised levels of restriction were slightly lower than the nominal rates^25^. Animals that are fed completely *ad libitum* (AL) may become obese and hence the comparison of CR to AL animals may simply reflect an anti-obesity effect of CR^15^,^30^. This is less of an issue when graded levels of CR are used instead of a single comparison of one CR level to AL animals. However, CR individuals will run out of food quickly and therefore the difference in food availability between a 24 h AL individual and a CR individual can impact the way these individuals will assort their activities throughout the day. To avoid these issues we used two ‘control groups’ exposed to 0% CR. For the first group (24AL) we allowed them 24 h access to food without restriction. For the second group (12AL) we allowed them unrestricted access to food for the 12 h of darkness but then removed the food at lights on (0630 h), replacing it 12 h later at lights off when the CR animals were also fed. Hence these animals, like the CR animals, did not have food available for prolonged period but were able to meet their energetic requirements over the 12-hour period when food was available to them. Water was continuously available to all individuals throughout the experiment. All animals were fed a high carbohydrate open source diet (D12450B: Research diets, NJ, USA) which contains 20% protein, 70% carbohydrate and 10% fat (by energy). Mice were housed individually and placed on antenna pads which allowed continuous triangulation of the position of the mice using surgically implanted transmitters (VitalViewTM telemetry and data acquisition system using a ER-4000 receiving platform (MiniMitter, OR, USA) (see^24^ for details). The tags were implanted intraperitoneally when mice were 12 weeks old. Data were recorded at minute intervals for the 2 weeks baseline period plus the entire 3 months of restriction. These tags also continuously sampled the mice body temperature. Using this information, we were able to observe the number of movements the mice made at given intervals (here every 15 minutes) as well as concurrently collect body temperature observations over the same intervals. A previous paper has addressed the patterns in the means of daily body temperature^26^. We used the sum of movements and the median body temperature over these 15 minute intervals as observations (one observation every 15 minutes for the 2 BL weeks and the 3 CR months). If the tag had a temporary failure during the 15-min interval, or the animal was temporarily removed from the cage, the sample was assigned a NA.

### Modelling approach

#### Model fitting

The fitting of these hidden Markov models to observations was implemented using depmixS4 1.3–2 31. This function derives the marginal log-likelihood of the observations and uses the expectation-maximisation (EM) to estimate parameters by maximizing the expected joint log-likelihood of the parameters given the observations and assumed states. This likelihood depends on the unobserved states S. We use unconstrained random seeds to draw initial parameter values and estimate initial S in the Expectation step. We then maximized the log-likelihood by maximizing separately for the prior parameters, transition parameters, and response parameters. The parameter estimation process was unconstrained. The same fitting process was used for all models.

#### Model selection

We selected best fitting models using the Akaike Information Criterion (AIC). Since we repeated this process for the control period when all mice were fed unrestricted, we could assess whether variability in phenotypic changes under CR was influenced by variability in phenotype before treatment commenced.

#### Model validation

Since the unobserved state of the mice cannot be known with certainty, it is difficult to estimate the residuals of each model capturing the overall goodness-of-fit of all model components. There exist a number of ways to estimate residuals, but all come with some caveats and a limited understanding of their expected distribution. Here we estimated the multivariate standardised pseudo-residuals^32^, post-fit. Given the variance structure of our multivariate function, we expect these residuals to be normally distributed. We visually inspected this assumption for model validation using quantile-quantile plots.

We did not include the effects of diurnal patterns in activity in the models which we know exists in mice. Our premise is that changes in activity associated with the light/dark phase in mice are part of the emergent properties of behavioural dynamics. We therefore expect to observe light/dark phase changes in activity budget to emerge from our model and use this as an independent, biologically relevant, test of the validity of our fitted models.

#### Model interpretation

We visualised state characteristics for each treatment level by simulating 1000 movement and body temperature from the fitted MVN for each mouse and each state to capture the shape of the MVN^33^. We tested for the effect of CR on phenotypic changes by fitting multivariate linear models for each state to individual fitted activity state characteristics (movement and temperature) in which CR level was an independent variable. We tested for changes in tpm between the BL and treatment period. Given the dependencies in tpm 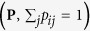 we fitted multivariate linear models for transitions from each state (models for p_1*j*_, p_2*j*_, and p_3*j*_ j ∈ [1; 2; 3]) to the paired difference in fitted tpm for individuals depending on their CR treatment, contrasting their BL and treatment tpm. We used Bonferroni adjustment for p-values to account for the multiple testing done in these multivariate linear models.

## Additional Information

**How to cite this article**: Lusseau, D. *et al.* The effects of graded levels of calorie restriction: IV. Non-linear change in behavioural phenotype of mice in response to short-term calorie restriction. *Sci. Rep.* **5**, 13198; doi: 10.1038/srep13198 (2015).

## Supplementary Material

Supplementary Information

## Figures and Tables

**Table 1:** Details of models fitted to mice observations:

**Model name**	**Model class**	Number ofstates	Effect ontpm	Effect onmovement	Effect ontemperature
HMM2.1	HMM1	2	–	–	–
HMM2.2	HMM2	2	–	t	–
HMM2.3	HMM2	2	–	–	t
HMM2.4	HMM2	2	–	t	t
HMM2.5	HMM3	2	–	BW	–
HMM2.6	HMM3	2	–	–	BW
HMM2.7	HMM3	2	–	BW	BW
HMM2.8	HMM4	2	t	–	–
HMM2.9	HMM5	2	BW	–	–
HMM2.10	HMM2+4	2	t	t	–
HMM2.11	HMM2+4	2	t	–	t
HMM2.12	HMM2+4	2	t	t	t
HMM2.13	HMM3+5	2	BW	BW	–
HMM2.14	HMM3+5	2	BW	–	BW
HMM2.15	HMM3+5	2	BW	BW	BW
HMM3.1	HMM1	3	–	–	–
HMM3.2	HMM2	3	–	t	–
HMM3.3	HMM2	3	–	–	t
HMM3.4	HMM2	3	–	t	t
HMM3.5	HMM3	3	–	BW	–
HMM3.6	HMM3	3	–	–	BW
HMM3.7	HMM3	3	–	BW	BW
HMM3.8	HMM4	3	t	–	–
HMM3.9	HMM5	3	BW	–	–
HMM3.10	HMM2+4	3	t	t	–
HMM3.11	HMM2+4	3	t	–	t
HMM3.12	HMM2+4	3	t	t	t
HMM3.13	HMM3+5	3	BW	BW	–
HMM3.14	HMM3+5	3	BW	–	BW
HMM3.15	HMM3+5	3	BW	BW	BW
t stands for the time elapsed since experiment started (scaled, in 15 minutes intervals), BW stands for daily body weight (scaled, in grams), tpm stands for transition probability matrix (**P**), and ‘–’ means that that effect was not included in that model.					
