# Supplementary material for: The effects of graded levels of calorie restriction: IV. Non-linear change in behavioural phenotype of mice in response to short-term calorie restriction
Source: Sci Rep. 2015 Aug 25;5:13198. doi: 10.1038/srep13198 (PMC4548231; doi:10.1038/srep13198)
Supplement: Supplementary Information [file srep13198-s1.pdf]

Non-linear change in behavioural phenotype of male C57BL/6 mice in response to short-term graded caloric restriction

Supplementary Information

David Lusseau[1†*], Sharon E. Mitchell[1†*], Ceres Barros[1], Davina Derous[1], Cara Green[1], Luonan Chen[2], Jing-Dong Jackie Han[3], Yingchun Wang[4], Daniel E.L. Promislow[5], Alex Douglas[1] and John R. Speakman[1,4*]

1. Institute of Biological and Environmental Sciences, University of Aberdeen, Aberdeen, Scotland, UK Ab24 2TZ

2. Key laboratory of Systems Biology, Shanghai Institute of Biological Sciences, Chinese Academy of Sciences, 800 Cao Bao road, Shanghai, China

3. Max Planck partner institute for computational biology , Shanghai

4. State Key laboratory of Molecular Developmental Biology, Institute of Genetics and Developmental Biology, Chinese Academy of Sciences, 1 Beichen Xilu, Chaoyang, Beijing, China

5. Department of Pathology, University of Washington at Seattle, Seattle, Washington, USA

[†]joint first authors

*: corresponding author: DL: d.lusseau@abdn.ac.uk; SEM: s.e.mitchell@abdn.ac.uk; JRS: j.speakman@abdn.ac.uk

List of supplementary information

Supplementary Table 1. Model selection for each individual to describe their behavioural phenotype during the baseline (BL) period. Values are ΔAIC, the difference in AIC between the most parsimonious model (the model with the smallest AIC, hence with a ΔAIC =0, highlighted in blue). ΔAIC provides information about how much support there is to consider alternative models to the most parsimonious one. Note the large ΔAIC for models with 2 states.

Supplementary Figure 1. Activity state characteristics for each treatment level at the end of the baseline period. The states are characterised by the log of the number of movements observed over a 15 minutes period and the median core body temperature of the mouse over those 15 minutes. Each colour represents a contour plot for a state (state 1 – blue, state 2 – red, state 3 - black) drawn from simulating 1000 observations for each mouse from its fitted HMM for each state. Simulated observations from all mice in a treatment level are cumulated in each contour plot. Note the high consistency within state between mice leading to well defined contours for each state.

Supplementary Table 2. Model selection for each individual to describe their behavioural phenotype during CR treatment. Values are ΔAIC, the difference in AIC between the most parsimonious model (the model with the smallest AIC, hence with a ΔAIC =0, highlighted in blue). ΔAIC provides information about how much support there is to consider alternative models to the most parsimonious one. Note the large ΔAIC for models with 2 states.

Supplementary Figure 2. Sample quantile-theoretical quantile plots for the final model of each mouse. Quantiles are derived from the standardised pseudo-residuals and expected to follow a normal distribution (solid line).

Supplementary Figure 3. Activity budget of mice throughout the CR treatment derived from the Viterbi sequence estimated from the posterior probabilities of activity state estimated by the best model for each 15-min interval for each mouse. Activity budgets are presented for the light and dark phases given the known diurnal cycle in mice activity. Note that the observed differences between these two phases were not explicitly incorporated in the model structure (Table 1) but instead emerge from the model fit.

Supplementary Table 1. Model selection for each individual to describe their behavioural phenotype during the baseline (BL) period. Values are ΔAIC, the difference in AIC between the most parsimonious model (the model with the smallest AIC, hence with a ΔAIC =0, highlighted in blue). ΔAIC provides information about how much support there is to consider alternative models to the most parsimonious one. Note the large ΔAIC for models with 2 states.

		**treatment**	**24AL**	**24AL**	**24AL**	**24AL**	**24AL**	**24AL**	**24AL**
model	model class	parameters	1	11	12	15	22	40	42
HMM2.1	HMM1	11	542.8	710.8	528.8	342.8	885.6	538.0	422.9
HMM2.2	HMM2	13	546.5	711.9	530.2	343.6	886.5	531.2	421.4
HMM2.3	HMM2	13	535.7	701.2	526.9	331.9	862.5	539.0	406.8
HMM2.4	HMM2	15	539.2	702.7	1565.2	333.1	864.2	1208.3	407.4
HMM2.5	HMM3	13	544.5	711.4	531.4	345.6	881.3	538.8	418.1
HMM2.6	HMM3	13	532.9	714.0	517.6	337.6	869.4	540.8	392.6
HMM2.7	HMM3	15	531.8	1164.4	1234.5	340.8	866.2	536.6	389.3
HMM2.8	HMM4	13	545.4	713.6	531.4	346.8	889.5	541.5	425.1
HMM2.9	HMM5	13	543.7	713.0	531.0	346.8	889.2	540.4	424.2
HMM2.10	HMM2+4	15	549.1	715.3	532.8	347.6	890.4	534.9	423.2
HMM2.11	HMM2+4	15	538.4	355.2	611.3	918.4	517.3	569.8	782.7
HMM2.12	HMM2+4	17	987.3	357.0	611.0	917.2	518.4	571.0	784.4
HMM2.13	HMM3+5	15	547.5	713.7	532.5	347.5	890.1	533.8	422.8
HMM2.14	HMM3+5	15	533.6	349.2	614.2	926.4	507.7	575.4	789.4
HMM2.15	HMM3+5	17	532.4	352.5	610.2	925.2	510.3	578.6	786.3
HMM3.1	HMM1	20	7.3	297.3	4.5	6.6	465.4	14.0	26.4
HMM3.2	HMM2	23	12.5	299.8	3.1	3.3	455.8	6.5	19.4
HMM3.3	HMM2	23	4.2	288.4	6.3	**0.0**	428.3	7.6	20.9
HMM3.4	HMM2	26	8.0	289.5	278.9	3.5	432.8	**0.0**	12.5
HMM3.5	HMM3	23	12.8	296.1	2.8	6.0	462.3	11.7	19.8
HMM3.6	HMM3	23	**0.0**	297.7	5.9	3.1	441.2	6.6	8.2
HMM3.7	HMM3	26	3.9	294.4	**0.0**	16.4	438.8	3.4	**0.0**
HMM3.8	HMM4	26	12.8	304.9	12.8	9.5	474.2	24.5	36.0
HMM3.9	HMM5	26	12.3	306.9	11.8	13.1	459.8	24.7	32.0
HMM3.10	HMM2+4	29	16.0	307.6	11.4	6.6	465.1	17.3	28.6
HMM3.11	HMM2+4	29	14.9	18.1	167.0	507.7	2.1	240.7	354.5
HMM3.12	HMM2+4	32	17.4	17.7	168.5	508.8	**0.0**	244.3	365.4
HMM3.13	HMM3+5	29	19.8	305.4	10.2	15.6	458.1	22.5	31.0
HMM3.14	HMM3+5	29	4.2	**0.0**	181.2	518.7	2.3	244.8	376.6
HMM3.15	HMM3+5	32	7.3	18.6	225.6	733.1	134.9	249.8	362.7

model	model class	treatment<br>parameters	12AL<br>16	12AL<br>17	12AL<br>18	12AL<br>20	12AL<br>31	12AL<br>32	12AL<br>38	12AL<br>45
HMM2.1	HMM1	11	1095	858	556	520	577	688	796	475
HMM2.2	HMM2	13	1099	857	558	522	582	691	650	474
HMM2.3	HMM2	13	1097	841	559	516	561	686	789	472
HMM2.4	HMM2	15	1100	1410	561	519	1091	690	760	470
HMM2.5	HMM3	13	1098	858	560	521	584	690	794	478
HMM2.6	HMM3	13	1099	850	559	505	561	686	769	468
HMM2.7	HMM3	15	1101	1554	563	504	1103	688	622	471
HMM2.8	HMM4	13	1099	858	556	523	579	690	799	479
HMM2.9	HMM5	13	1099	861	560	523	579	691	797	478
HMM2.10	HMM2+4	15	1103	858	558	526	583	693	650	478
HMM2.11	HMM2+4	15	532	406	641	600	586	510	1004	870
HMM2.12	HMM2+4	17	522	406	1272	1170	1230	513	1005	873
HMM2.13	HMM3+5	15	1103	860	561	526	584	694	652	477
HMM2.14	HMM3+5	15	532	390	642	611	590	499	1003	871
HMM2.15	HMM3+5	17	1095	387	645	611	593	497	1006	872
HMM3.1	HMM1	20	549	501.4	17.4	35.5	50.4	177.6	9.5	8.9
HMM3.2	HMM2	23	548	506.9	14.7	41.4	53.3	181.1	4.2	7.3
HMM3.3	HMM2	23	548	470.6	2.5	21.8	5.1	178.5	1.6	**0.0**
HMM3.4	HMM2	26	548	465.0	**0.0**	334.0	**0.0**	382.6	294.9	0.2
HMM3.5	HMM3	23	544	507.1	18.0	33.5	54.4	178.6	2.7	13.0
HMM3.6	HMM3	23	551	483.9	17.7	**0.0**	18.9	145.8	13.6	3.6
HMM3.7	HMM3	26	1038	485.3	21.1	244.4	24.5	149.7	6.8	5.7
HMM3.8	HMM4	26	555	508.7	21.2	47.0	62.0	187.2	13.4	15.9
HMM3.9	HMM5	26	555	503.2	26.2	47.1	56.6	186.0	7.2	18.1
HMM3.10	HMM2+4	29	555	514.3	22.1	52.9	62.5	190.5	8.4	13.5
HMM3.11	HMM2+4	29	7.3	27.7	99.1	236.6	36.4	23.4	455.6	369.5
HMM3.12	HMM2+4	32	**0.0**	17.2	101.3	229.4	359.2	28.7	718.2	509.7
HMM3.13	HMM3+5	29	555.3	509.0	22.1	45.0	61.4	186.6	**0.0**	22.0
HMM3.14	HMM3+5	29	6.0	9.6	102.2	246.9	52.6	**0.0**	467.7	331.1
HMM3.15	HMM3+5	32	2.1	**0.0**	98.0	249.6	51.7	368.4	473.3	333.7

model	model class	treatment parameters	10CR 8	10CR 9	10CR 21	10CR 33	10CR 46	10CR 50	10CR 54	10CR 56
HMM2.1	HMM1	11	975	862	544	445	442	316	695	714.0
HMM2.2	HMM2	13	971	866	542	446	444	315	697	711.3
HMM2.3	HMM2	13	965	861	547	375	442	300	693	697.2
HMM2.4	HMM2	15	961	864	545	375	445	300	695	1226.7
HMM2.5	HMM3	13	974	865	545	442	438	320	688	714.8
HMM2.6	HMM3	13	959	862	547	415	438	319	697	710.6
HMM2.7	HMM3	15	958	864	547	416	434	671	687	983.0
HMM2.8	HMM4	13	979	865	545	448	445	319	699	716.5
HMM2.9	HMM5	13	979	866	548	448	445	320	699	716.9
HMM2.10	HMM2+4	15	974	869	542	450	448	318	701	712.9
HMM2.11	HMM2+4	15	785	466	393	726	1146	711	436	287.4
HMM2.12	HMM2+4	17	756	454	927	729	1144	711	439	288.2
HMM2.13	HMM3+5	15	975	869	546	450	448	319	700	714.2
HMM2.14	HMM3+5	15	764	462	387	728	1149	750	432	308.1
HMM2.15	HMM3+5	17	618	774	385	1100	1150	751	428	312.0
HMM3.1	HMM1	20	582.4	358.5	150.0	98.4	8.5	131.2	271.2	182.3
HMM3.2	HMM2	23	574.1	366.0	134.4	103.3	13.5	14.0	272.5	184.1
HMM3.3	HMM2	23	587.9	358.2	149.3	**0.0**	6.4	**0.0**	262.8	153.6
HMM3.4	HMM2	26	564.3	359.1	130.3	3.5	10.1	197.8	510.8	152.8
HMM3.5	HMM3	23	585.7	360.5	151.5	86.1	11.0	136.8	261.9	176.0
HMM3.6	HMM3	23	565.1	353.3	329.9	58.0	**0.0**	124.0	269.8	176.3
HMM3.7	HMM3	26	566.2	538.4	154.8	376.0	242.3	256.9	262.2	170.9
HMM3.8	HMM4	26	589.0	375.9	146.2	104.8	19.9	19.4	280.4	192.6
HMM3.9	HMM5	26	592.8	370.1	160.1	117.9	18.1	16.7	282.0	191.7
HMM3.10	HMM2+4	29	581.7	372.4	142.1	118.2	24.9	24.1	281.2	194.2
HMM3.11	HMM2+4	29	**0.0**	**0.0**	28.2	232.0	748.3	338.1	38.1	122.9
HMM3.12	HMM2+4	32	479.2	287.6	7.1	235.9	734.8	341.2	12.4	**0.0**
HMM3.13	HMM3+5	29	596.2	377.0	161.8	96.6	20.7	19.0	272.6	185.8
HMM3.14	HMM3+5	29	4.8	4.1	**0.0**	230.5	759.9	396.3	**0.0**	6.7
HMM3.15	HMM3+5	32	17.4	3.8	1.4	350.4	761.8	529.7	257.3	9.3

model	model class	**treatment** parameters	**20CR** 4	**20CR** 10	**20CR** 27	**20CR** 37	**20CR** 39	**20CR** 47	**20CR** 57	**20CR** 64
HMM2.1	HMM1	11	812.7	739.7	620.1	293.0	508.9	560.4	645.1	434.1
HMM2.2	HMM2	13	814.2	735.1	623.7	276.0	512.0	563.5	645.0	432.5
HMM2.3	HMM2	13	813.9	733.7	623.9	281.5	512.4	563.5	646.1	432.5
HMM2.4	HMM2	15	815.1	731.0	1071	268.5	515.5	566.6	646.8	430.1
HMM2.5	HMM3	13	815.8	743.0	620.3	296.8	508.8	559.8	627.6	434.3
HMM2.6	HMM3	13	806.6	738.3	620.1	291.4	509.4	563.2	636.2	436.4
HMM2.7	HMM3	15	808.9	741.8	621.4	295.2	509.4	560.8	611.8	436.8
HMM2.8	HMM4	13	816.0	738.8	623.6	296.1	512.9	564.3	648.8	436.6
HMM2.9	HMM5	13	813.2	742.7	623.4	295.3	511.9	563.7	648.5	438.0
HMM2.10	HMM2+4	15	817.0	735.3	627.2	279.2	516.0	567.4	648.8	435.0
HMM2.11	HMM2+4	15	425.1	537.3	468.4	903.0	995.9	275.0	507.6	622.9
HMM2.12	HMM2+4	17	426.9	517.6	468.8	901.2	999.4	262.2	510.7	625.9
HMM2.13	HMM3+5	15	814.5	738.2	627.1	278.3	515.0	566.8	648.3	436.4
HMM2.14	HMM3+5	15	428.6	551.2	457.8	912.6	991.9	284.2	503.6	621.7
HMM2.15	HMM3+5	17	418.0	550.2	459.6	916.2	993.2	288.0	627.9	619.0
HMM3.1	HMM1	20	342.7	285.6	281.4	36.5	16.8	47.1	341.4	**0.0**
HMM3.2	HMM2	23	339.4	270.5	285.9	**0.0**	20.3	45.1	342.1	2.5
HMM3.3	HMM2	23	345.6	279.8	277.7	39.4	16.4	49.3	345.8	0.5
HMM3.4	HMM2	26	340.6	257.4	280.2	40.3	246.2	50.6	342.1	283.6
HMM3.5	HMM3	23	343.1	289.4	283.6	40.5	13.7	47.8	336.1	0.4
HMM3.6	HMM3	23	337.1	285.7	271.2	35.2	6.8	48.6	308.9	5.3
HMM3.7	HMM3	26	728.2	278.8	275.0	182.0	**0.0**	47.7	436.5	4.8
HMM3.8	HMM4	26	349.8	289.8	292.6	44.7	23.0	54.5	352.0	8.4
HMM3.9	HMM5	26	350.7	295.9	291.5	45.4	18.3	55.7	357.2	14.9
HMM3.10	HMM2+4	29	347.4	277.1	297.0	6.3	26.8	54.2	365.3	10.6
HMM3.11	HMM2+4	29	**0.0**	1.0	7.9	457.0	657.1	35.3	14.4	111.9
HMM3.12	HMM2+4	32	1.4	**0.0**	1.2	677.3	791.6	**0.0**	24.6	112.6
HMM3.13	HMM3+5	29	357.5	300.0	293.4	48.2	15.9	57.8	347.3	10.1
HMM3.14	HMM3+5	29	8.2	23.5	**0.0**	471.0	649.9	33.2	**0.0**	112.3
HMM3.15	HMM3+5	32	1.0	616.9	7.9	461.3	773.0	49.8	70.6	113.2

		**treatment**	**30CR**	**30CR**	**30CR**	**30CR**	**30CR**	**30CR**	**30CR**
model	model class	parameters	6	24	36	49	52	53	63
HMM2.1	HMM1	11	665.5	973.5	323.5	327.8	358.8	458.1	396.2
HMM2.2	HMM2	13	654.8	973.9	311.3	330.4	362.7	455.3	393.4
HMM2.3	HMM2	13	664.7	968.2	318.2	318.2	349.2	461.5	398.0
HMM2.4	HMM2	15	649.3	968.7	614.4	321.6	351.3	459.0	395.4
HMM2.5	HMM3	13	655.1	974.2	306.9	330.6	360.2	450.4	399.5
HMM2.6	HMM3	13	661.6	971.3	327.4	307.5	358.8	460.7	389.3
HMM2.7	HMM3	15	650.7	1600	590.2	306.0	359.7	454.3	392.9
HMM2.8	HMM4	13	668.0	971.6	325.9	330.9	357.9	461.4	399.9
HMM2.9	HMM5	13	669.1	975.5	327.3	331.5	362.8	461.2	397.7
HMM2.10	HMM2+4	15	657.3	972.6	313.6	333.5	361.6	458.4	397.2
HMM2.11	HMM2+4	15	330.9	426.8	781.3	770.7	406.1	312.9	443.3
HMM2.12	HMM2+4	17	815.1	424.6	765.8	771.9	390.4	316.3	446.3
HMM2.13	HMM3+5	15	658.6	976.1	315.2	333.9	366.6	458.6	394.8
HMM2.14	HMM3+5	15	320.7	432.2	779.5	778.2	416.0	302.6	456.7
HMM2.15	HMM3+5	17	296.5	432.5	768.7	778.6	396.8	300.5	457.6
HMM3.1	HMM1	20	186.5	369.4	29.9	60.0	10.1	67.7	27.7
HMM3.2	HMM2	23	169.5	365.8	14.9	11.1	11.9	56.5	28.4
HMM3.3	HMM2	23	181.0	361.8	**0.0**	62.6	**0.0**	57.8	31.9
HMM3.4	HMM2	26	158.5	348.3	143.5	74.5	193.6	64.7	218.9
HMM3.5	HMM3	23	184.5	368.6	7.1	65.8	2.7	50.8	21.1
HMM3.6	HMM3	23	184.1	364.5	27.3	**0.0**	11.7	66.9	**0.0**
HMM3.7	HMM3	26	184.1	628.4	184.6	2.6	196.6	48.9	3.6
HMM3.8	HMM4	26	196.1	380.3	41.0	19.9	15.0	76.8	36.1
HMM3.9	HMM5	26	193.3	385.0	37.6	68.0	11.8	66.9	32.1
HMM3.10	HMM2+4	29	181.8	377.4	23.7	18.2	16.7	63.9	37.1
HMM3.11	HMM2+4	29	37.0	**0.0**	303.5	177.6	95.2	6.8	100.6
HMM3.12	HMM2+4	32	185.8	2.1	281.3	164.0	134.0	**0.0**	348.2
HMM3.13	HMM3+5	29	194.1	383.2	14.1	73.7	4.1	57.9	24.8
HMM3.14	HMM3+5	29	**0.0**	277.8	307.4	178.9	119.2	51.5	107.1
HMM3.15	HMM3+5	32	97.5	12.2	569.8	181.3	263.4	56.9	105.2

model	model class	treatment<br>parameters	40CR<br>7	40CR<br>28	40CR<br>30	40CR<br>34	40CR<br>44	40CR<br>48	40CR<br>58	40CR<br>62
HMM2.1	HMM1	11	639.2	913.1	461.8	415.8	471.1	358.4	402.4	536.1
HMM2.2	HMM2	13	617.7	915.1	462.3	414.9	471.1	356.0	404.8	539.6
HMM2.3	HMM2	13	634.4	908.0	439.7	414.9	447.3	357.5	403.1	527.3
HMM2.4	HMM2	15	610.0	1394	432.9	411.8	436.9	356.3	404.8	831.7
HMM2.5	HMM3	13	639.2	912.9	459.1	419.4	474.7	360.9	404.0	534.9
HMM2.6	HMM3	13	632.2	912.8	461.8	418.0	472.1	354.4	396.6	530.3
HMM2.7	HMM3	15	628.1	912.0	451.3	741.7	473.2	356.6	399.9	526.2
HMM2.8	HMM4	13	643.1	916.8	462.2	417.7	475.1	362.2	405.5	539.1
HMM2.9	HMM5	13	642.7	915.6	462.5	418.6	474.1	360.8	406.0	539.3
HMM2.10	HMM2+4	15	621.3	918.8	462.4	415.8	474.4	359.8	408.0	542.7
HMM2.11	HMM2+4	15	409.0	397.2	567.5	708.0	509.7	691.5	440.6	330.4
HMM2.12	HMM2+4	17	406.1	394.6	542.6	710.1	503.2	1071	861.8	764.5
HMM2.13	HMM3+5	15	621.4	917.6	462.4	417.8	474.5	358.4	408.4	542.9
HMM2.14	HMM3+5	15	408.0	386.8	564.8	711.3	529.0	695.7	465.1	325.6
HMM2.15	HMM3+5	17	402.4	756.9	560.4	710.6	520.3	698.4	582.9	327.9
HMM3.1	HMM1	20	103.8	398.1	29.6	15.1	22.8	42.6	62.9	87.5
HMM3.2	HMM2	23	61.0	393.5	33.1	0.6	24.3	45.7	67.1	90.1
HMM3.3	HMM2	23	97.3	384.4	19.1	14.0	**0.0**	28.7	59.1	75.1
HMM3.4	HMM2	26	51.7	692.3	268.9	279.7	1.4	**0.0**	259.7	346.3
HMM3.5	HMM3	23	105.6	390.9	33.2	17.6	28.2	47.5	68.0	89.2
HMM3.6	HMM3	23	105.5	374.0	19.1	15.8	23.8	13.1	41.7	90.5
HMM3.7	HMM3	26	106.7	363.3	34.4	22.6	210.9	42.4	45.9	192.6
HMM3.8	HMM4	26	113.2	406.6	34.5	15.5	33.3	20.7	72.9	98.8
HMM3.9	HMM5	26	112.7	399.0	38.0	25.3	32.6	50.4	70.5	97.8
HMM3.10	HMM2+4	29	71.2	402.8	37.9	**0.0**	34.7	54.3	77.2	101.0
HMM3.11	HMM2+4	29	22.7	35.8	36.6	188.7	91.7	288.2	**0.0**	4.4
HMM3.12	HMM2+4	32	17.2	46.0	**0.0**	186.9	90.5	276.4	1.7	207.1
HMM3.13	HMM3+5	29	113.4	391.7	41.7	27.8	37.8	24.6	82.8	99.4
HMM3.14	HMM3+5	29	21.5	**0.0**	44.0	169.8	95.0	300.7	24.0	21.0
HMM3.15	HMM3+5	32	**0.0**	3.4	45.3	158.7	97.1	302.1	29.2	**0.0**

Supplementary Figure 1. Activity state characteristics for each treatment level at the end of the baseline period. The states are characterised by the log of the number of movements observed over a 15 minutes period and the median core body temperature of the mouse over those 15 minutes. Each colour represents a contour plot for a state (state 1 – blue, state 2 – red, state 3 - black) drawn from simulating 1000 observations for each mouse from its fitted HMM for each state. Simulated observations from all mice in a treatment level are cumulated in each contour plot. Note the high consistency within state between mice leading to well defined contours for each state.

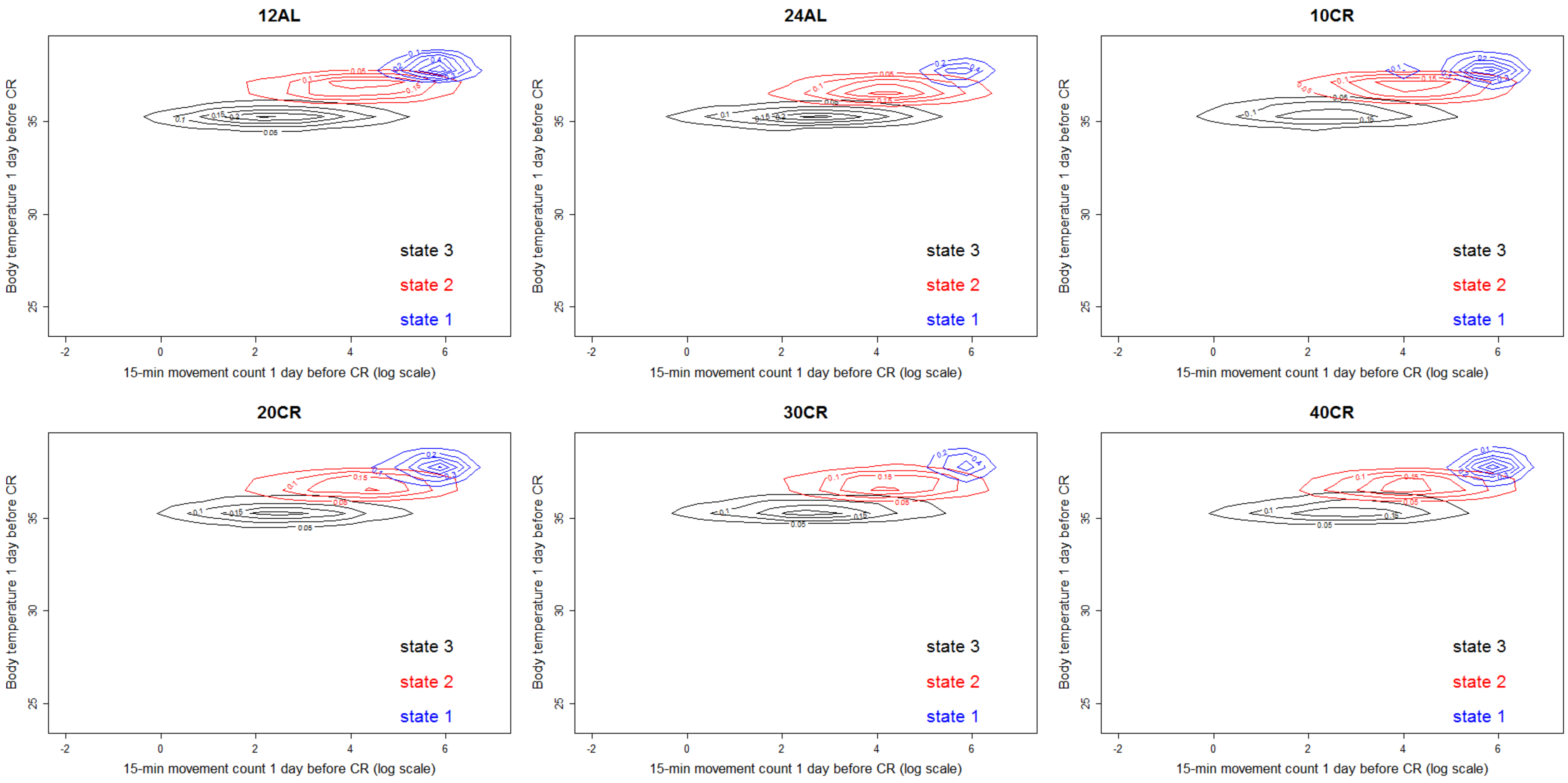

Supplementary Table 2. Model selection for each individual to describe their behavioural phenotype during CR treatment. Values are ΔAIC, the difference in AIC between the most parsimonious model (the model with the smallest AIC, hence with a ΔAIC =0, highlighted in blue). ΔAIC provides information about how much support there is to consider alternative models to the most parsimonious one. Note the large ΔAIC for models with 2 states.

model	model class	**treatment** parameters	**24AL** 1	**24AL** 11	**24AL** 12	**24AL** 13	**24AL** 15	**24AL** 22	**24AL** 40	**24AL** 42
HMM2.1	HMM1	11	4015.5	2639.5	4970.1	7512.2	3361.0	3651.2	3227.8	3207.0
HMM2.2	HMM2	13	4005.5	2631.7	4961.0	7511.9	3313.6	3620.3	3205.4	3196.5
HMM2.3	HMM2	13	3771.5	2498.4	4912.6	7361.0	2970.3	3465.8	3100.5	3119.2
HMM2.4	HMM2	15	3758.2	2495.9	4903.0	7347.4	2914.0	3430.1	3041.1	3109.6
HMM2.5	HMM3	13	4010.2	2633.9	4962.5	7494.3	3305.5	3629.6	3223.3	3201.7
HMM2.6	HMM3	13	3974.3	2513.6	4933.0	6891.7	3133.8	3492.3	3219.8	3095.4
HMM2.7	HMM3	15	3972.1	2509.9	4929.1	6848.6	3095.3	3472.8	3214.1	3090.0
HMM2.8	HMM4	13	3975.7	2629.6	4971.5	7512.9	3328.9	3624.2	3201.2	3174.9
HMM2.9	HMM5	13	3971.7	2628.1	4970.3	7511.9	3328.6	3614.8	3211.3	3174.2
HMM2.10	HMM2+4	15	3968.0	2622.1	4963.1	7513.8	3284.3	3599.1	3183.6	3166.5
HMM2.11	HMM2+4	15	3727.6	2492.2	4914.6	7364.4	2947.7	3445.5	3075.4	3088.1
HMM2.12	HMM2+4	17	3718.1	2490.8	4905.9	7351.3	2896.7	3415.6	3024.3	3080.8
HMM2.13	HMM3+5	15	3964.3	2620.4	4961.8	7511.6	3284.1	3588.5	3191.9	3165.5
HMM2.14	HMM3+5	15	3930.1	2508.8	4933.1	6892.5	3104.8	3459.3	3203.5	3064.2
HMM2.15	HMM3+5	17	3930.9	2506.6	4929.9	6848.0	3070.3	3444.5	3200.2	3061.1
HMM3.1	HMM1	20	379.0	123.4	57.5	281.3	462.5	204.5	148.3	297.7
HMM3.2	HMM2	23	365.9	96.6	47.6	285.1	404.2	190.3	146.3	292.7
HMM3.3	HMM2	23	59.0	**0.0**	5.3	3842.8	99.1	25.0	40.2	285.4
HMM3.4	HMM2	26	43.0	0.2	**0.0**	13.0	34.1	**0.0**	21.0	276.4
HMM3.5	HMM3	23	372.5	105.5	40.9	281.9	391.0	176.1	148.7	299.1
HMM3.6	HMM3	23	279.9	24.7	25.0	33.1	243.4	58.3	143.4	248.9
HMM3.7	HMM3	26	278.9	29.9	16.7	15.7	189.3	42.7	143.3	30.0
HMM3.8	HMM4	26	344.9	107.0	60.1	264.0	421.7	172.6	127.1	269.8
HMM3.9	HMM5	26	341.9	106.2	61.1	282.4	426.1	168.7	130.1	260.5
HMM3.10	HMM2+4	29	331.9	76.3	50.1	268.3	356.7	161.0	126.2	263.7
HMM3.11	HMM2+4	29	8.6	4.4	9.9	3851.9	60.5	1.8	15.3	260.2
HMM3.12	HMM2+4	32	**0.0**	1227.3	5.3	**0.0**	**0.0**	1954.9	**0.0**	249.5
HMM3.13	HMM3+5	29	335.3	81.0	44.3	283.8	359.2	143.1	131.7	260.2
HMM3.14	HMM3+5	29	234.3	29.6	28.8	36.6	202.1	31.9	125.3	8.8
HMM3.15	HMM3+5	32	237.4	34.3	20.8	20.7	152.3	20.3	128.0	**0.0**

model	model class	treatment parameters	12AL 16	12AL 17	12AL 18	12AL 20	12AL 31	12AL 32	12AL 38	12AL 45
HMM2.1	HMM1	11	4316.5	3943.5	4482.0	3850.5	3563	4129	4019	4111
HMM2.2	HMM2	13	4297.7	3924.0	4363.8	3818.7	3544	4126	4012	4111
HMM2.3	HMM2	13	4268.5	3912.3	4277.4	3785.4	3524	4010	3975	4010
HMM2.4	HMM2	15	4246.6	3892.1	4153.4	3748.7	3507	3997	3967	7906
HMM2.5	HMM3	13	4304.2	3912.1	4410.1	3820.9	3559	4131	4010	4104
HMM2.6	HMM3	13	4220.8	3863.6	4222.0	3776.7	3557	4117	3849	4038
HMM2.7	HMM3	15	4209.4	3831.1	4151.4	3744.6	3552	9556	3839	4026
HMM2.8	HMM4	13	4303.1	3937.3	4470.4	3839.9	3554	4132	4001	4082
HMM2.9	HMM5	13	4272.5	3934.0	4463.9	3832.5	3543	4132	3974	4095
HMM2.10	HMM2+4	15	4285.0	3919.5	4356.0	3809.5	3537	4129	3996	4084
HMM2.11	HMM2+4	15	4254.5	3906.9	4266.4	3776.4	3515	4013	3959	3987
HMM2.12	HMM2+4	17	4233.4	12050	4146.9	3741.0	3500	4000	3953	7895
HMM2.13	HMM3+5	15	4254.5	3916.3	4349.1	3802.3	3527	4129	3969	4096
HMM2.14	HMM3+5	15	4178.5	3856.0	4204.1	3760.4	3538	4120	3807	4024
HMM2.15	HMM3+5	17	4169.5	3825.9	4138.3	11151	3534	4121	8699	4015
HMM3.1	HMM1	20	87.2	27.1	354.8	126.9	118.4	151.6	673.4	291.5
HMM3.2	HMM2	23	87.0	17.4	286.1	120.3	67.8	156.2	270.6	293.3
HMM3.3	HMM2	23	68.0	31.7	148.5	38.6	21.3	**0.0**	600.8	47.2
HMM3.4	HMM2	26	60.4	22.4	24.8	**0.0**	8.9	815.2	580.7	4.2
HMM3.5	HMM3	23	90.9	11.3	320.7	116.8	125.6	156.6	673.6	278.9
HMM3.6	HMM3	23	24.6	19.7	98.2	73.1	87.2	136.1	77.4	152.2
HMM3.7	HMM3	26	17.1	4.6	6.9	43.0	77.8	136.2	564.8	91.9
HMM3.8	HMM4	26	67.8	25.8	353.5	124.9	137.9	1212.4	267.8	286.7
HMM3.9	HMM5	26	62.2	21.2	338.9	125.8	116.0	158.6	237.2	288.5
HMM3.10	HMM2+4	29	70.8	15.7	270.9	116.9	71.0	158.8	671.5	289.7
HMM3.11	HMM2+4	29	48.3	29.9	139.2	50.9	24.6	4.9	592.5	45.6
HMM3.12	HMM2+4	32	2264.7	20.4	**0.0**	8.6	**0.0**	815.5	569.6	**0.0**
HMM3.13	HMM3+5	29	65.6	5.9	310.4	113.2	90.7	163.4	643.2	278.3
HMM3.14	HMM3+5	29	**0.0**	14.1	90.2	78.7	82.6	145.3	33.2	155.2
HMM3.15	HMM3+5	32	1.8	**0.0**	4.6	46.7	76.0	145.5	**0.0**	95.2

model	model class	treatment parameters	10CR 8	10CR 9	10CR 21	10CR 33	10CR 46	10CR 50	10CR 54	10CR 56
HMM2.1	HMM1	11	4294	4350	4546	3730	4603	3893	3136	4545
HMM2.2	HMM2	13	4263	4346	4547	3690	4591	3859	3138	4531
HMM2.3	HMM2	13	4273	4056	4534	3597	4508	3785	2940	4455
HMM2.4	HMM2	15	7877	8853	4535	9068	10590	3752	2940	4443
HMM2.5	HMM3	13	4298	4353	4532	3706	4581	3873	3137	4527
HMM2.6	HMM3	13	3853	3540	4504	3600	4584	3784	3114	4364
HMM2.7	HMM3	15	3851	3531	4491	3576	4556	3771	3116	11063
HMM2.8	HMM4	13	4298	4349	4523	3704	4605	3858	3115	4529
HMM2.9	HMM5	13	4295	4350	4535	3705	4606	3897	3140	4544
HMM2.10	HMM2+4	15	4266	4344	4524	3668	4594	3827	3118	4517
HMM2.11	HMM2+4	15	4276	4056	4510	3573	4511	3751	2919	4438
HMM2.12	HMM2+4	17	7879	8855	4511	9057	4488	3721	2919	4428
HMM2.13	HMM3+5	15	4264	4346	4536	3668	4595	3862	3142	4531
HMM2.14	HMM3+5	15	3855	3543	4493	3577	4587	3788	3118	4361
HMM2.15	HMM3+5	17	3854	8934	4482	3556	4559	3775	8646	4353
HMM3.1	HMM1	20	449.4	1648.5	76.5	165.3	336.6	163.4	211.3	219.0
HMM3.2	HMM2	23	420.0	1597.2	67.9	168.4	285.1	165.8	192.3	192.4
HMM3.3	HMM2	23	524.5	1188.5	31.5	81.3	92.7	153.6	28.0	136.7
HMM3.4	HMM2	26	416.9	6964.0	9.3	7.4	19.0	154.5	23.6	84.4
HMM3.5	HMM3	23	451.3	1580.4	69.7	155.5	318.2	142.1	192.2	198.5
HMM3.6	HMM3	23	68.6	587.5	63.0	79.8	313.2	4.7	213.0	7.4
HMM3.7	HMM3	26	**0.0**	8.0	51.6	10.5	293.1	**0.0**	168.5	**0.0**
HMM3.8	HMM4	26	453.0	1641.7	73.3	139.4	319.5	125.9	185.3	213.7
HMM3.9	HMM5	26	525.3	1651.0	73.7	147.4	340.2	171.0	211.8	220.7
HMM3.10	HMM2+4	29	500.1	1592.4	62.8	144.0	266.3	130.0	172.5	178.6
HMM3.11	HMM2+4	29	529.6	1180.1	26.4	67.8	71.8	119.5	11.5	129.3
HMM3.12	HMM2+4	32	421.7	1026.1	**0.0**	5.0	**0.0**	121.8	**0.0**	79.7
HMM3.13	HMM3+5	29	449.4	1587.2	66.1	140.9	322.6	150.1	199.6	201.1
HMM3.14	HMM3+5	29	73.8	45.3	61.1	71.8	313.5	12.6	173.3	9.2
HMM3.15	HMM3+5	32	1.1	**0.0**	49.0	**0.0**	293.8	1409.2	167.3	10.3

model	model class	treatment parameters	20CR 4	20CR 10	20CR 27	20CR 37	20CR 39	20CR 47	20CR 57	20CR 64
HMM2.1	HMM1	11	5062	4360	4272	3816	4655	4170	3943	3772
HMM2.2	HMM2	13	5009	4348	4249	3798	4652	4164	3896	3765
HMM2.3	HMM2	13	4972	4348	4061	3572	4575	3806	3856	2926
HMM2.4	HMM2	15	4941	10603	4048	3523	8831	8541	3796	8262
HMM2.5	HMM3	13	5058	4358	4267	3811	4645	4167	3946	3755
HMM2.6	HMM3	13	4965	4257	3771	3601	4348	3707	3712	3069
HMM2.7	HMM3	15	4966	9582	3752	3577	4344	3702	6316	3058
HMM2.8	HMM4	13	5059	4356	4274	3820	4631	4162	3942	3772
HMM2.9	HMM5	13	5063	4360	4273	3818	4650	4166	3943	3767
HMM2.10	HMM2+4	15	5005	4344	4250	3801	4630	4155	3897	3763
HMM2.11	HMM2+4	15	4971	4345	4064	3576	4550	3805	3853	2924
HMM2.12	HMM2+4	17	11611	4334	4055	9933	4548	3806	3795	2906
HMM2.13	HMM3+5	15	5010	4348	4250	3799	4647	4160	3897	3758
HMM2.14	HMM3+5	15	4966	4257	3774	3604	4344	3709	3712	3066
HMM2.15	HMM3+5	17	4967	4250	3754	3579	4341	3704	3709	3055
HMM3.1	HMM1	20	475.0	327.5	859.6	402.5	207.9	603.0	468.3	1113.6
HMM3.2	HMM2	23	459.1	323.2	777.1	296.0	208.5	524.1	393.2	993.9
HMM3.3	HMM2	23	11.3	218.1	494.9	154.4	192.0	204.6	343.9	21.7
HMM3.4	HMM2	26	5.0	217.2	464.1	64.1	190.0	203.2	265.8	1459.0
HMM3.5	HMM3	23	468.4	323.7	743.6	334.5	269.6	533.5	436.7	1017.8
HMM3.6	HMM3	23	153.9	19.3	75.4	65.1	11.6	17.7	26.6	253.1
HMM3.7	HMM3	26	148.3	**0.0**	15.3	6.9	70.6	21.0	13.3	242.6
HMM3.8	HMM4	26	465.6	324.0	853.7	405.7	251.7	591.3	465.7	1111.8
HMM3.9	HMM5	26	468.4	335.0	849.4	403.2	199.3	601.9	460.7	1105.0
HMM3.10	HMM2+4	29	452.2	319.7	768.5	289.2	188.3	506.4	402.0	981.4
HMM3.11	HMM2+4	29	4.6	194.8	477.3	144.3	219.1	187.2	351.8	27.1
HMM3.12	HMM2+4	32	**0.0**	193.8	1870.7	47.6	207.3	185.7	275.4	**0.0**
HMM3.13	HMM3+5	29	461.0	329.7	732.4	328.4	249.6	527.0	434.6	992.5
HMM3.14	HMM3+5	29	151.5	22.2	62.2	64.7	6.2	7.0	32.5	247.0
HMM3.15	HMM3+5	32	146.7	2.0	**0.0**	**0.0**	**0.0**	**0.0**	**0.0**	236.3

model	model class	treatment parameters	30CR 6	30CR 24	30CR 36	30CR 49	30CR 52	30CR 53	30CR 63
HMM2.1	HMM1	11	4957	4682	3285	3250	6490	4095	2922
HMM2.2	HMM2	13	4955	4681	3285	3196	6490	4087	2843
HMM2.3	HMM2	13	4368	4626	3235	3146	6036	3536	2468
HMM2.4	HMM2	15	4353	4621	3238	3102	6004	3522	2427
HMM2.5	HMM3	13	4953	4657	3256	3156	6492	4092	2815
HMM2.6	HMM3	13	4259	4134	3101	2981	5743	3299	2214
HMM2.7	HMM3	15	4225	4106	4865	2915	5705	3275	2145
HMM2.8	HMM4	13	4949	4685	3269	3235	6478	4089	2897
HMM2.9	HMM5	13	4957	4682	3263	3232	6487	4092	2900
HMM2.10	HMM2+4	15	4945	4684	3268	3178	6480	4082	2788
HMM2.11	HMM2+4	15	4356	4630	3215	3132	6035	3526	2449
HMM2.12	HMM2+4	17	4340	4625	7576	3085	6007	3513	2400
HMM2.13	HMM3+5	15	4955	4681	3262	3175	6487	4085	2791
HMM2.14	HMM3+5	15	4254	4135	3080	2968	5745	3297	2204
HMM2.15	HMM3+5	17	4217	4107	4824	2896	5708	7464	2135
HMM3.1	HMM1	20	903.6	937.8	741.5	368.6	1125.4	1018.3	557.4
HMM3.2	HMM2	23	874.7	933.5	739.3	368.4	1053.0	951.7	509.4
HMM3.3	HMM2	23	191.0	846.2	508.4	552.6	638.8	327.9	149.3
HMM3.4	HMM2	26	180.2	842.2	478.4	477.3	575.4	328.2	129.8
HMM3.5	HMM3	23	888.9	914.0	708.8	391.2	1107.2	930.6	488.1
HMM3.6	HMM3	23	13.0	39.7	79.0	417.6	64.8	13.2	139.3
HMM3.7	HMM3	26	**0.0**	**0.0**	**0.0**	300.8	30.4	5.6	111.9
HMM3.8	HMM4	26	876.8	946.3	510.0	463.5	1116.2	1013.3	490.7
HMM3.9	HMM5	26	874.2	913.3	732.3	330.9	1092.8	1008.9	468.7
HMM3.10	HMM2+4	29	848.2	942.1	733.1	353.8	1044.6	945.7	449.9
HMM3.11	HMM2+4	29	191.3	855.4	492.5	263.3	644.9	313.5	57.9
HMM3.12	HMM2+4	32	1852.3	851.0	461.8	216.3	581.3	314.1	37.9
HMM3.13	HMM3+5	29	858.1	887.9	696.5	346.9	1075.5	917.2	406.2
HMM3.14	HMM3+5	29	12.5	48.1	60.6	**0.0**	34.6	**0.0**	41.2
HMM3.15	HMM3+5	32	1738.7	8.3	396.5	354.0	**0.0**	523.0	**0.0**

model	model class	treatment parameters	**40CR** 7	**40CR** 28	**40CR** 30	**40CR** 34	**40CR** 44	**40CR** 48	**40CR** 58	**40CR** 62
HMM2.1	HMM1	11	6109	6333	7952	7139	5742	6181	7183	7391
HMM2.2	HMM2	13	6102	6292	7890	7023	5720	6182	7171	7377
HMM2.3	HMM2	13	5987	5592	7605	5783	4776	5935	6959	6948
HMM2.4	HMM2	15	5964	5564	7501	5703	4750	5930	6861	6864
HMM2.5	HMM3	13	6107	6267	7911	6993	5702	6163	7130	7377
HMM2.6	HMM3	13	5725	5207	7359	5711	4158	5529	6908	6700
HMM2.7	HMM3	15	5724	5149	8148	6334	4094	6027	6873	6580
HMM2.8	HMM4	13	6109	6335	7944	7128	5742	6172	7174	7393
HMM2.9	HMM5	13	6099	6330	7952	7112	5740	6177	7174	7389
HMM2.10	HMM2+4	15	6103	6295	7878	7011	5719	6172	7165	7379
HMM2.11	HMM2+4	15	5989	5594	7604	5786	4771	5903	6949	6943
HMM2.12	HMM2+4	17	5967	5564	7497	5704	4742	5891	6848	6856
HMM2.13	HMM3+5	15	6094	6290	7890	6988	5717	6176	7164	7377
HMM2.14	HMM3+5	15	5721	5207	7362	5715	4158	5531	6902	6704
HMM2.15	HMM3+5	17	5721	5152	7202	5645	4092	5425	6866	6583
HMM3.1	HMM1	20	432.7	1084.1	1057.1	2029.6	1414.9	798.8	181.4	1183.5
HMM3.2	HMM2	23	420.3	978.8	981.5	1913.9	1241.0	787.5	162.7	1165.0
HMM3.3	HMM2	23	315.4	438.2	680.1	109.3	617.7	442.9	81.2	661.9
HMM3.4	HMM2	26	325.0	428.6	595.3	85.9	597.5	418.6	54.9	591.7
HMM3.5	HMM3	23	432.9	989.9	1027.5	1942.7	1212.5	767.3	142.2	1185.9
HMM3.6	HMM3	23	8.8	47.9	133.1	44.9	237.9	114.0	25.0	103.9
HMM3.7	HMM3	26	7.4	34.0	11.9	1211.1	191.0	54.5	11.8	25.2
HMM3.8	HMM4	26	440.8	1068.7	1053.2	2016.6	1358.4	808.4	189.8	1178.6
HMM3.9	HMM5	26	426.3	1056.4	1041.0	1978.7	1224.7	725.3	178.0	1158.4
HMM3.10	HMM2+4	29	427.9	961.1	975.2	1902.2	1167.6	797.2	159.4	1159.1
HMM3.11	HMM2+4	29	326.0	429.7	683.7	109.3	594.2	451.9	79.9	657.3
HMM3.12	HMM2+4	32	3998.8	420.4	597.0	84.3	570.8	427.9	54.0	586.1
HMM3.13	HMM3+5	29	426.6	958.0	1011.0	1892.3	1020.7	690.5	125.5	1160.2
HMM3.14	HMM3+5	29	2.6	16.2	121.9	9.9	68.0	58.5	14.3	79.2
HMM3.15	HMM3+5	32	**0.0**	**0.0**	**0.0**	**0.0**	**0.0**	**0.0**	**0.0**	**0.0**

Supplementary Figure 2. Sample quantile-theoretical quantile plots for the final model of each mouse. Quantiles are derived from the standardised pseudo-residuals and expected to follow a normal distribution (solid line).

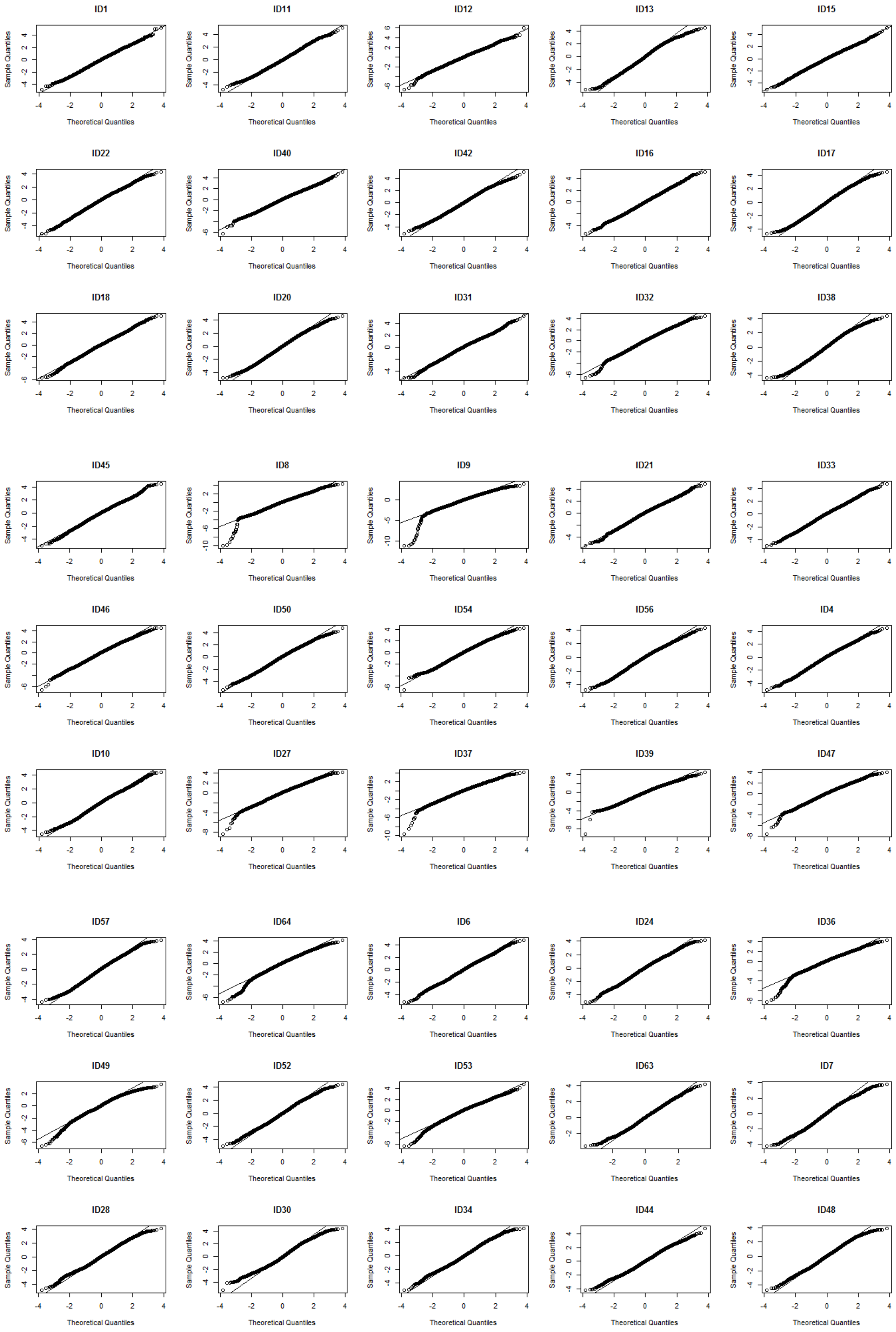

Supplementary Figure 3. Activity budget of mice throughout the CR treatment derived from the Viterbi sequence estimated from the posterior probabilities of activity state estimated by the best model for each 15-min interval for each mouse. Activity budgets are presented for the light and dark phases given the known diurnal cycle in mice activity. Note that the observed differences between these two phases were not explicitly incorporated in the model structure (Table 1) but instead emerge from the model fit.

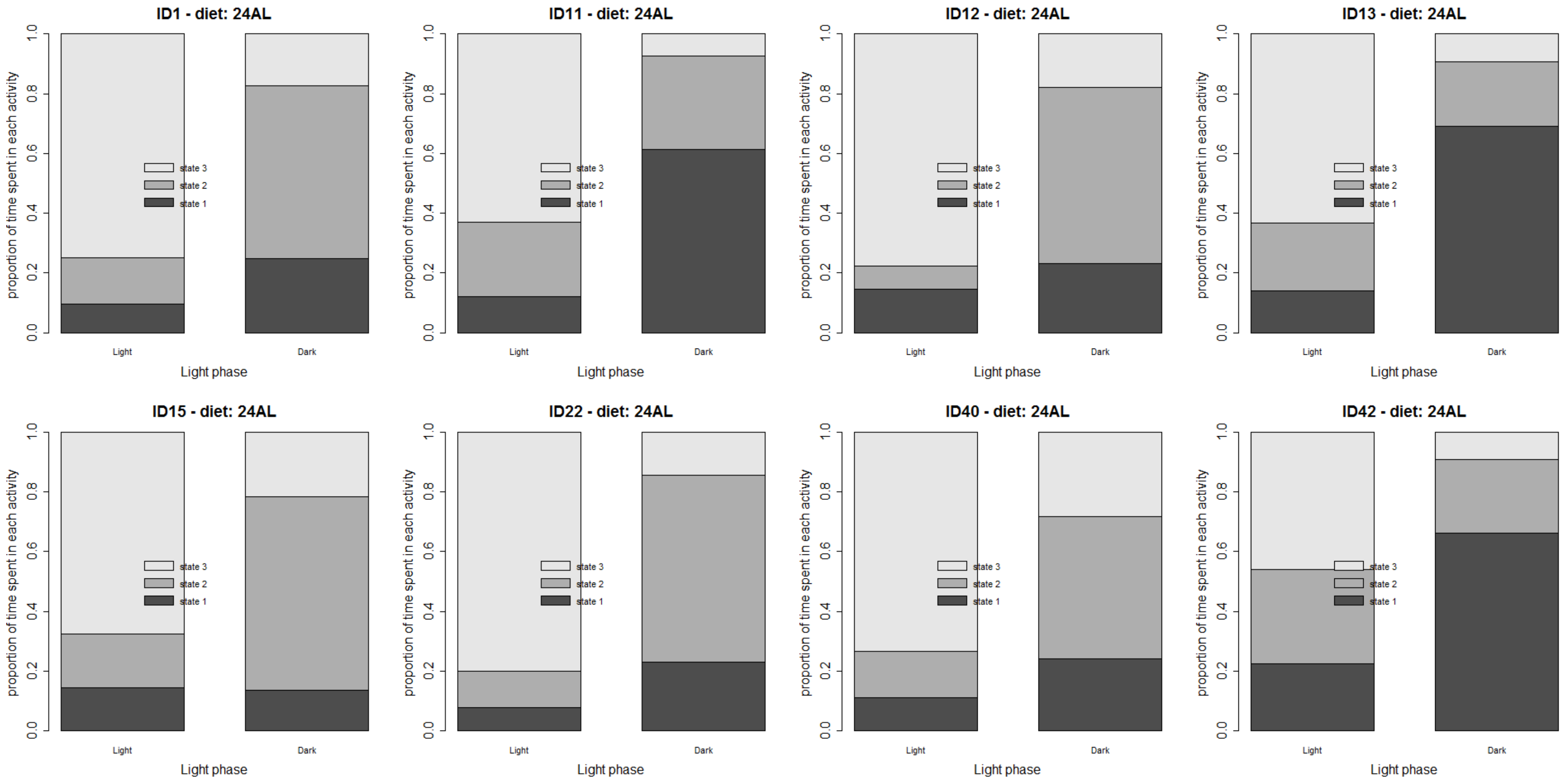

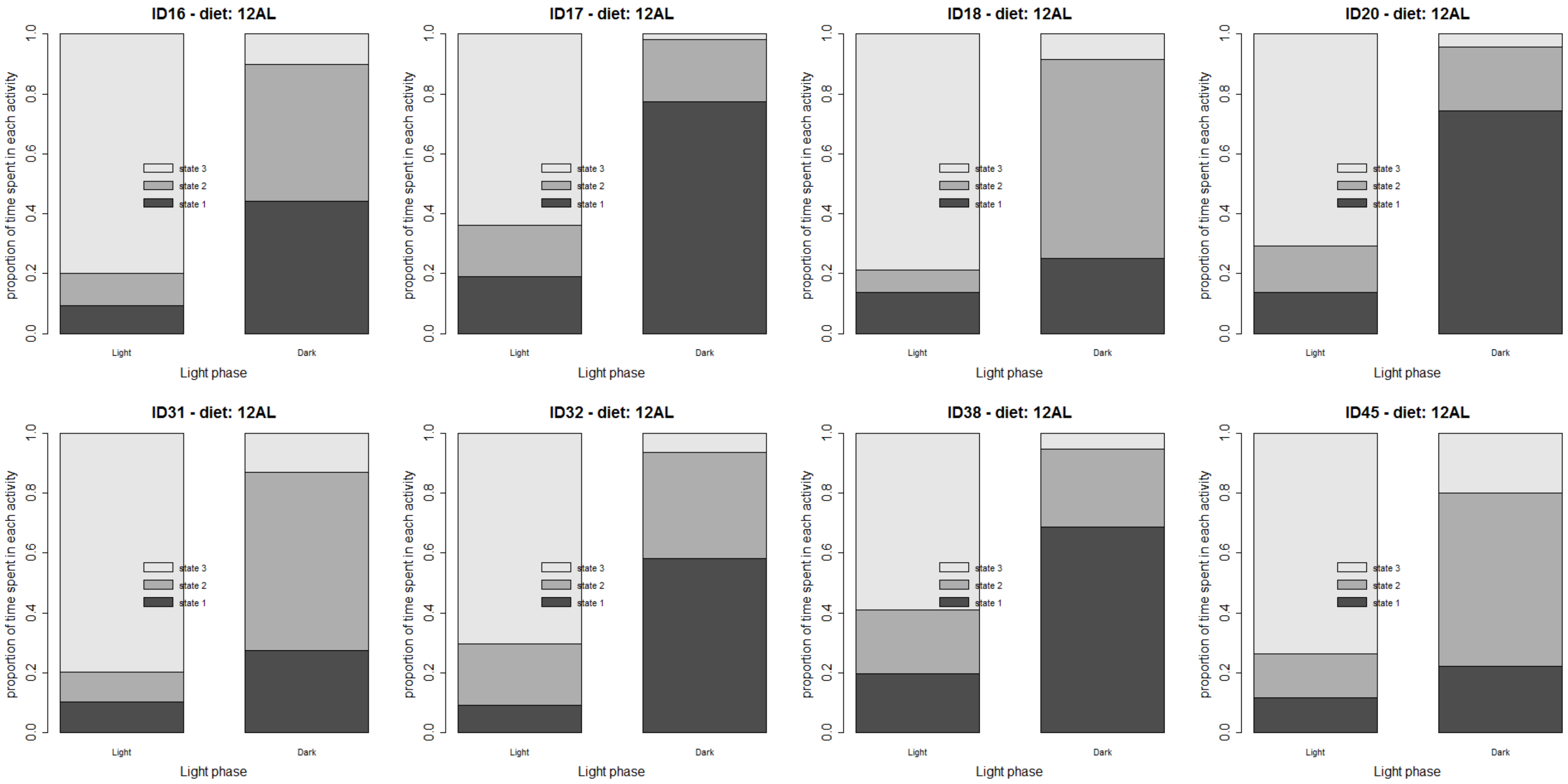
ID16 - diet: 12AL
ID17 - diet: 12AL
ID18 - diet: 12AL
ID20 - diet: 12AL
ID31 - diet: 12AL
ID32 - diet: 12AL
ID38 - diet: 12AL
ID45 - diet: 12AL
proportion of time spent in each activity
0.0
0.2
0.4
0.6
0.8
1.0
state 3
state 2
state 1
Light
Dark
Light phase

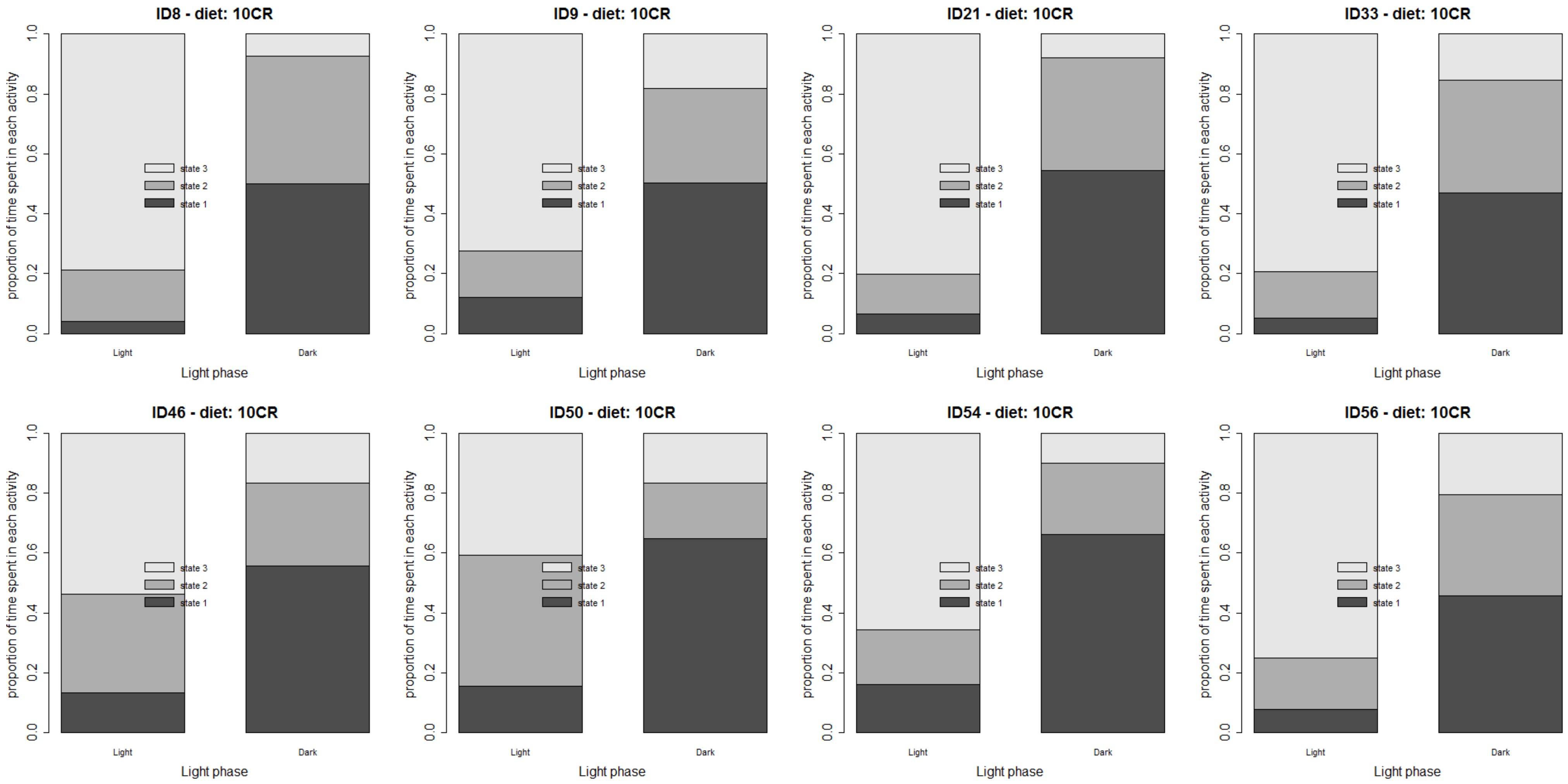

ID8 - diet: 10CR
ID9 - diet: 10CR
ID21 - diet: 10CR
ID33 - diet: 10CR
ID46 - diet: 10CR
ID50 - diet: 10CR
ID54 - diet: 10CR
ID56 - diet: 10CR
proportion of time spent in each activity
0.0
0.2
0.4
0.6
0.8
1.0
state 3
state 2
state 1
Light
Dark
Light phase

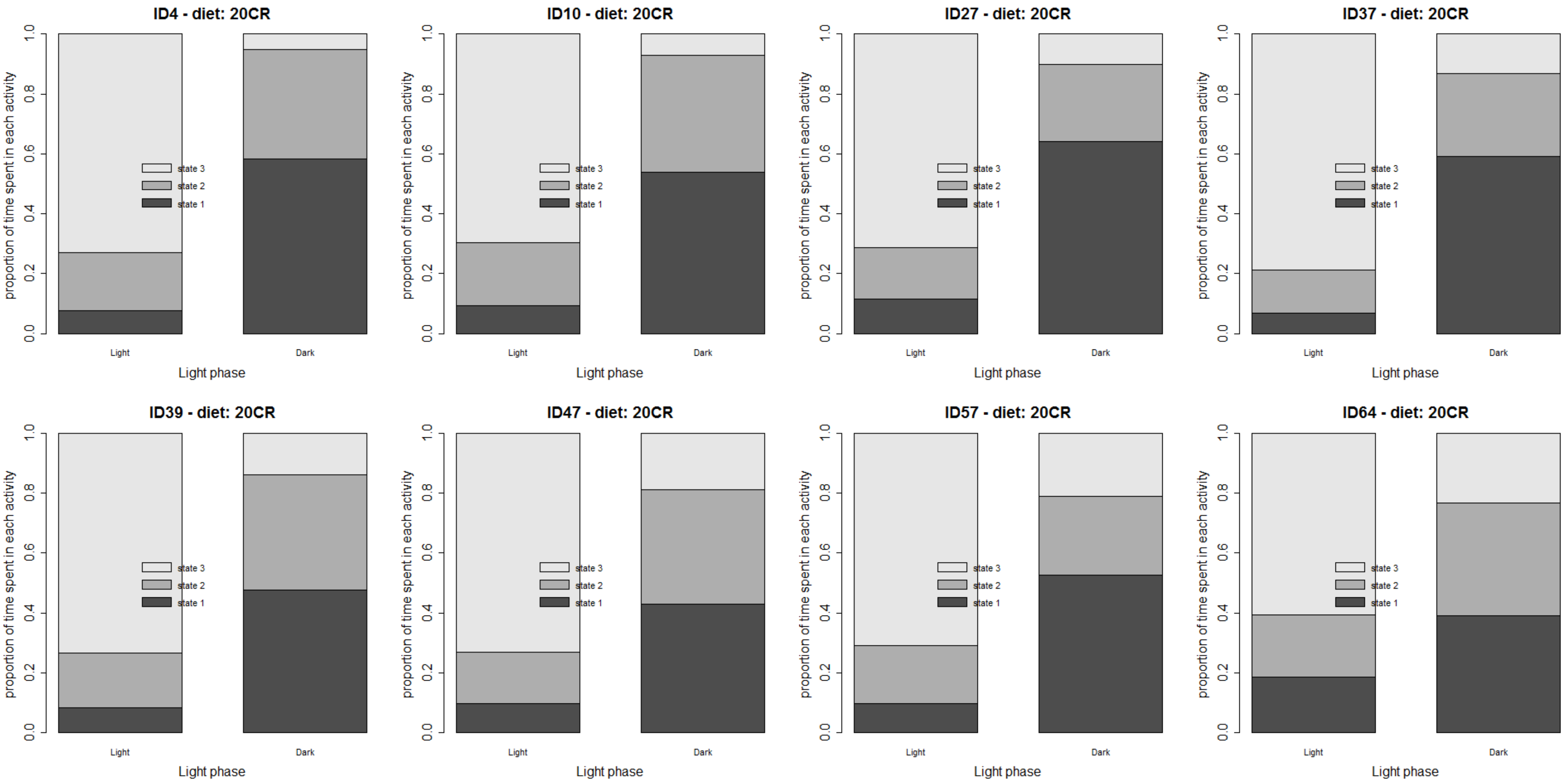
ID4 - diet: 20CR
ID10 - diet: 20CR
ID27 - diet: 20CR
ID37 - diet: 20CR
ID39 - diet: 20CR
ID47 - diet: 20CR
ID57 - diet: 20CR
ID64 - diet: 20CR
proportion of time spent in each activity
0.0
0.2
0.4
0.6
0.8
1.0
state 3
state 2
state 1
Light
Dark
Light phase

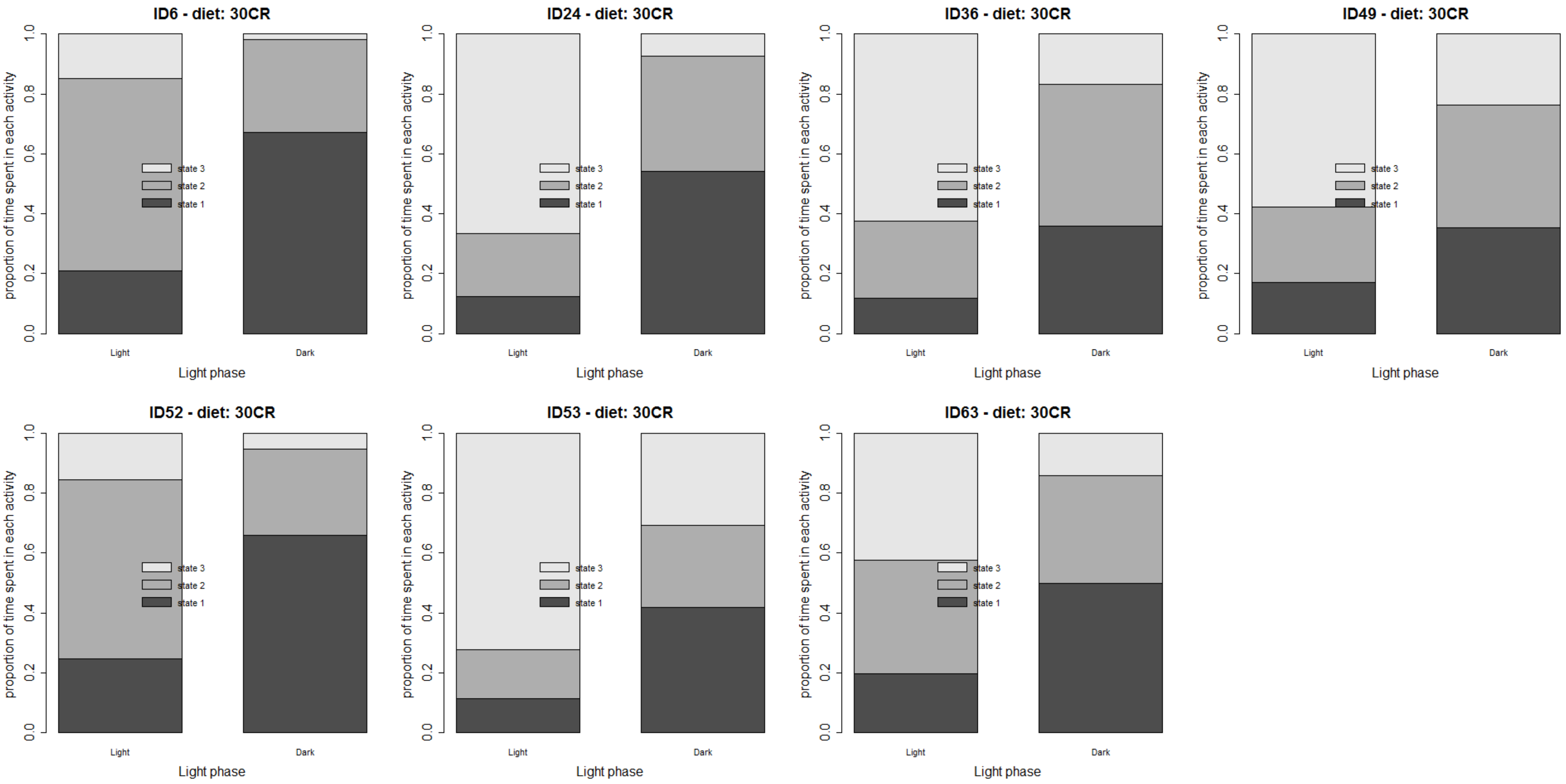
ID6 - diet: 30CR
ID24 - diet: 30CR
ID36 - diet: 30CR
ID49 - diet: 30CR
ID52 - diet: 30CR
ID53 - diet: 30CR
ID63 - diet: 30CR
proportion of time spent in each activity
0.0
0.2
0.4
0.6
0.8
1.0
state 3
state 2
state 1
Light
Dark
Light phase

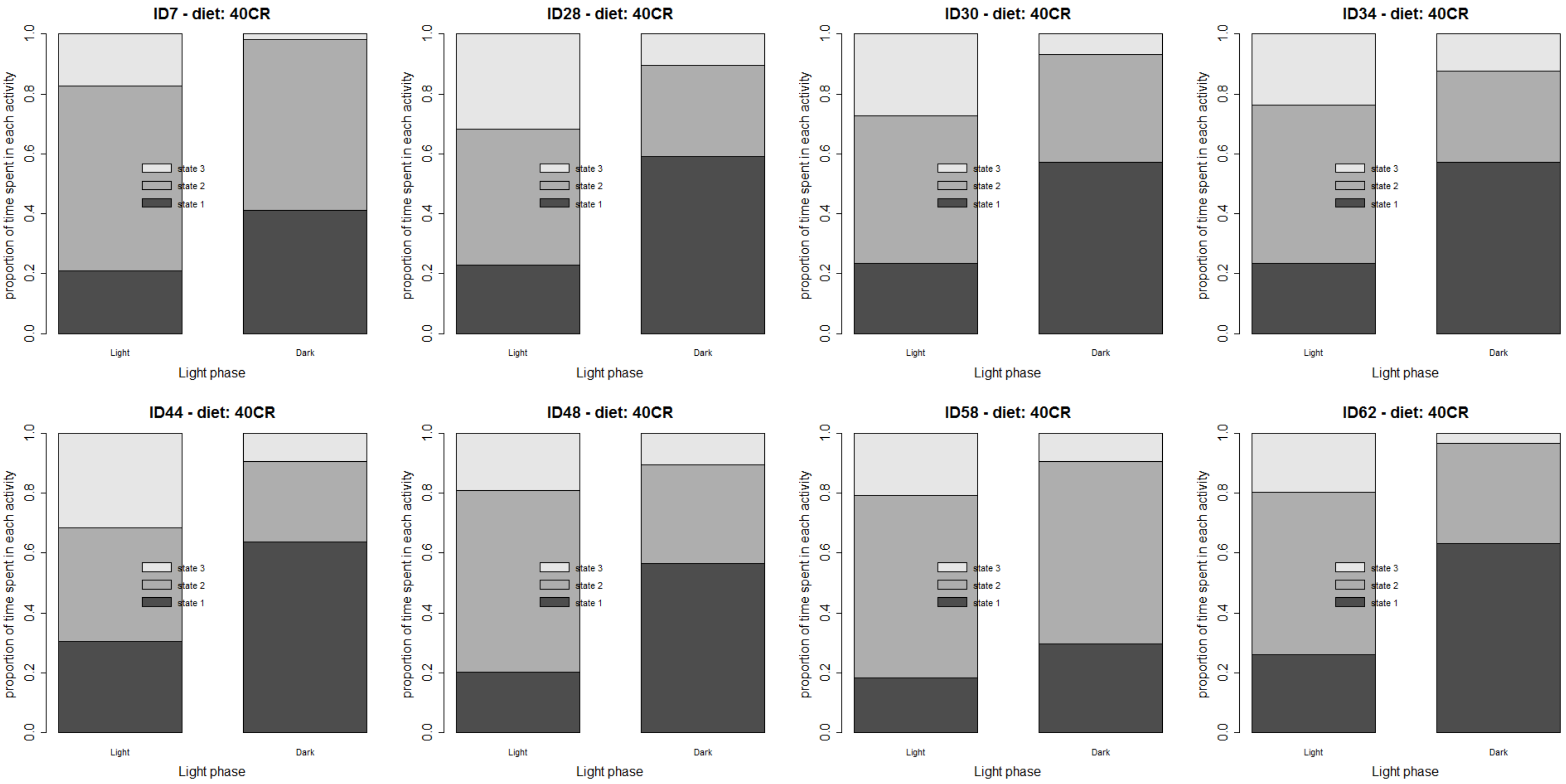
ID7 - diet: 40CR
ID28 - diet: 40CR
ID30 - diet: 40CR
ID34 - diet: 40CR
ID44 - diet: 40CR
ID48 - diet: 40CR
ID58 - diet: 40CR
ID62 - diet: 40CR
proportion of time spent in each activity
0.0
0.2
0.4
0.6
0.8
1.0
state 3
state 2
state 1
Light
Dark
Light phase
